# Psychometric Considerations in Assessing Fear Generalization as a Predictor of Anxiety

**DOI:** 10.1016/j.bpsgos.2025.100570

**Published:** 2025-07-25

**Authors:** Yannik Stegmann, Mario Reutter, Katharina Hutterer, Lea Hildebrandt, Jürgen Deckert, Lorenz Deserno, Katharina Domschke, Tina B. Lonsdorf, Paul Pauli, Andreas Reif, Karoline Rosenkranz, Miriam A. Schiele, Dirk Schümann, Peter Zwanzger, Marta Andreatta, Matthias Gamer

**Affiliations:** a Department of Psychology, University of Würzburg, Würzburg, Germany; b Department of Psychiatry, Psychosomatics and Psychotherapy, Center of Mental Health, University Hospital Würzburg, Würzburg, Germany; c Department of Child and Adolescent Psychiatry, Psychotherapy and Psychosomatics, Center for Mental Health, University Hospital Würzburg, Würzburg, Germany; d Max PIanck Institute for Human Cognitive and Brain Sciences, Leipzig, Germany; e Department of Psychiatry and Psychotherapy, Technische Universität Dresden, Dresden, Germany; f Department of Psychiatry and Psychotherapy, Medical Center – University of Freiburg, Faculty of Medicine, University of Freiburg, Freiburg, Germany; g Institute for Systems Neuroscience, University Medical Center Hamburg-Eppendorf, Hamburg, Germany; h Department of Psychology, Biological Psychology and Cognitive Neuroscience, University of Bielefeld, Bielefeld, Germany; i Department of Psychiatry, Psychosomatic Medicine and Psychotherapy, Goethe University Frankfurt, University Hospital, Frankfurt, Germany; j Fraunhofer Institute for Translational Medicine and Pharmacology, Frankfurt, Germany; k kbo-Inn-Salzach-Klinikum, Clinical Center for Psychiatry, Psychotherapy, Psychosomatic Medicine, Geriatrics and Neurology, Wasserburg am Inn, Germany; l Department of Psychiatry, Ludwig-Maximilian-University Munich, Munich, Germany; m Department of General Psychiatry and Psychotherapy, University Hospital Tübingen, Tübingen, Germany; n Tübingen Center for Mental Health, University Hospital Tübingen, Tübingen, Germany

**Keywords:** Anxiety, Fear, Generalization, Learning, Reliability, Threat

## Abstract

**Background:**

The ability to adaptively transfer acquired fear to novel situations is fundamental for survival in ever-changing environments and may contribute to the emergence and persistence of anxiety disorders. Consequently, research has focused on the assessment of fear generalization profiles to predict individual differences in anxiety. However, substantial heterogeneity in the operationalization of generalization hampers comparisons across studies and poses a risk to the replicability of findings.

**Methods:**

To address these issues, we reviewed the literature to identify commonly used methods for characterizing perceptual fear generalization profiles. Then, we conducted simulation analyses to examine correlations between indices and probe their robustness against measurement noise. Finally, we used 2 large empirical datasets (*N* = 1175 and *N* = 256 healthy humans) to examine the reliability of these indices and their validity in predicting anxiety-related traits.

**Results:**

All identified indices were substantially correlated but highly sensitive to measurement noise, with only minimal differences between methods. Reliabilities were moderate for subjective ratings but poor for skin conductance responses. All indices of fear generalization were unrelated to anxiety-related traits.

**Conclusions:**

Overall, a more comprehensive discussion of conceptual and methodological issues is needed to enable informed decisions about how to reliably and validly estimate fear generalization and its relationship with anxiety-related traits or clinical symptoms.

## Plain Language Summary

Understanding how fear generalizes to new situations is key to explaining individual differences in anxiety, but current studies vary widely in how generalization is measured. We reviewed existing methods and used both simulated and real data to examine their reliability and validity. While the measures were strongly related, none consistently predicted individual anxiety levels. These findings highlight the need for clearer conceptual frameworks and more robust measurement approaches in future research on fear generalization.

Fear and anxiety are emotional states that help individuals cope with threats or adverse situations. However, when these emotions occur unselectively across different situations, are exaggerated and difficult to manage, and result in widespread avoidance behavior, they become maladaptive and mark the transition to an anxiety disorder (1). Being the most prevalent mental disorders in Western countries (2, 3, 4), anxiety disorders generate substantial costs for society due to the reduced ability to work among affected individuals, their difficulties in establishing and maintaining social relationships, and the risk of suicide (5).

Given the importance of detecting the risk for anxiety disorders early and providing effective treatment, research has focused on identifying psychological and neural mechanisms that contribute to the etiology and maintenance of such clinical conditions. Related efforts have frequently relied on laboratory tasks to characterize aberrant aversive associations (6), such as Pavlovian fear conditioning (7). In these tasks, participants are typically confronted with one stimulus that is paired with an aversive experience (unconditioned stimulus [US]) and thereby becomes a conditioned stimulus (CS+). In differential fear conditioning protocols, another stimulus is presented but never paired with the aversive US and thereby becomes a CS that signals safety (CS−). This paradigm has been extensively used to experimentally model the development of anxiety disorders in healthy individuals (8, 9, 10, 11). Patients with anxiety disorders show an exaggerated fear response to the CS− that results in decreased CS differentiation [for a meta-analysis, see (12)]. Thus, both highly anxious but healthy individuals (13) as well as patients with anxiety disorders (14) seem to generalize fear to stimuli that were never associated with threat before.

Fear generalization was already described more than a century ago in the seminal study of “little Albert” (15), who was initially conditioned to associate a white rat (CS+) with an aversive sound (US) and later showed fear responses to stimuli that had perceptual similarity to CS+ (e.g., rabbit, fur coat, cotton wool). Recently, interest surged when differences in perceptual fear generalization between healthy individuals and patients with anxiety disorders were observed (14,16, 17, 18, 19). These studies frequently relied on a fear acquisition procedure but included generalization stimuli (GS) (e.g., circles of different sizes) (19) that covered the similarity continuum between CS+ (e.g., large circle) and CS− (e.g., small circle). These paradigms typically result in generalization gradients that span from CS− to CS+, capturing the extent to which fear responses generalize across perceptual similarity. Several studies have shown that these gradients tend to be more linear for patients with anxiety but show a substantial curvature in healthy participants, with only those GS most similar to the CS+ eliciting increased fear responses (14,19).

Although recent meta-analyses have confirmed significant relationships between the extent of fear generalization and anxious personality traits (13) as well as pathological anxiety (20,21), they have also reported significant heterogeneity across studies. This might have resulted from a lack of a gold standard to quantify the extent of fear generalization on an individual level from fear generalization profiles. Numerous indices to quantify individual fear generalization profiles have been proposed, but their psychometric properties, which are key for the prediction of individual differences (22,23), remain largely unexplored. This is problematic because individualized treatments, which have been proposed as a viable alternative to manualized psychotherapy (24,25), critically rely on the accurate measurement of potentially maladaptive processes. Such heterogeneity in measurements not only hampers the quantification of treatment effects but also limits scientific progress by reducing replicability (26).

Therefore, our goals were to first identify commonly used indices for characterizing fear generalization profiles by reviewing the available literature. Second, we described the psychometric properties of these indices, their robustness against measurement noise, and their interrelations based on simulation data. Finally, we used 2 large datasets (*N* = 1175 and *N* = 256), including ratings and electrodermal responses, to estimate the split-half reliability of these indices as well as their validity in predicting individual differences in anxiety.

## Methods and Materials

### Literature Review of Methods for Characterizing Fear Generalization Profiles

**Figure 1:**
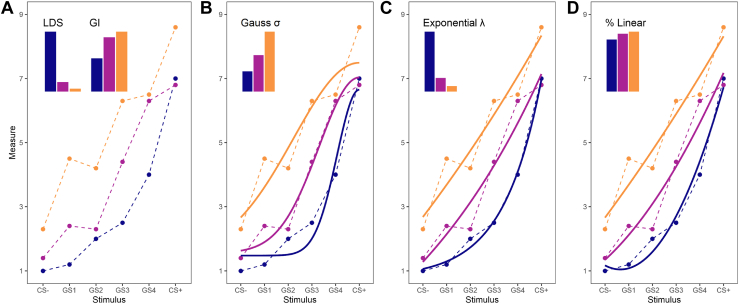
Comparison of arithmetic solutions **(A)** and model-fitting approaches **(B–D)** for quantifying the strength of generalization in 3 schematic participants. Exemplary fear generalization data for 3 different participants are depicted as blue, purple, and orange dots connected by dashed lines. Solid curved lines illustrate the fitted models described by the respective parameter values. **(A)** Linear deviation score (LDS): The LDS can be illustrated as a measure of how strongly the responses to the generalization stimuli (GS) deviate from a hypothetical straight line between the responses to conditioned stimuli (CS− and CS+). Values near 0 indicate a linear gradient, whereas negative values indicate stronger generalization, respectively. Generalization index (GI): The GI can be considered as the combined response strength to the GS relative to the fear stimulus. Higher values indicate stronger fear generalization. **(B)** Gaussian model fit (σ_Gauss_): The Gaussian function has 3 free parameters: the standard deviation of the normal distribution σ_Gauss_, the scale factor *b*_Gauss_, and the vertical translation factor *a*_Gauss_. Thus, σ_Gauss_ is the best single parameter to describe the curvature of the generalization gradient, with higher values indicating more generalization. **(C)** Exponential model fit: the exponential function is characterized by the growth factor exponential model fit (λ_exp_), the scale factor *n*_exp_, and the vertical translation factor *a*_exp_. The exponential growth factor λ_exp_ is primarily responsible for the curvature of the function and therefore most accurately describes the strength of generalization. Higher values indicate reduced generalization. **(D)** Regression model fit: analogous to the linear and quadratic trends in the analysis of variance, individual fear generalization parameters can be obtained by fitting a polynomial function including a quadratic, linear, and constant term. To combine the weights of the quadratic and linear terms into a single parameter, the relative importance of the linear term (%_linquad_) over the quadratic term can be calculated, which is expressed as the relative contribution of the linear term to the total explained variance. For LDS and λ_exp_, high values indicate less fear generalization.

To identify commonly calculated generalization indices, we conducted a systematic literature review, including 115 records in the analysis (see the Supplement for details). Most studies applied 3 different procedures to assess the profile of fear generalization (see Table 1): 1) 14 studies relied on arithmetic solutions to calculate a generalization index, and the most common were the linear deviation score (LDS) and the generalization index (GI); 2) 37 studies considered linear and quadratic trends resulting from analysis of variance (ANOVA) models; and 3) 30 studies modeled the generalization gradient using Gaussian or exponential functions (see Figure 1 for an overview of the indices and the Supplement for an in-depth description). To summarize, our review shows large analytic heterogeneity in characterizing fear generalization profiles. Most studies focused on group-level differences (e.g., considering linear and quadratic trends within ANOVA models) without calculating indices of fear generalization on an individual level.

**Table 1:** Laboratory Measures of Fear Generalization

Studies That Arithmetically Calculated an Index to Quantify Fear Generalization	
LDS	Aslanidou *et al.* (56), Imholze *et al.* (41), Kaczkurkin *et al.* (57), Lange *et al.* (58), Lange *et al.* (31), Lissek *et al.* (14), Reutter *et al.* (59), Stegmann *et al.* (27), Zhu *et al.* (60)
GI	Herzog *et al.* (61), Lenaert *et al.* (31), Mertens *et al.* (62), Reinhard *et al.* (63), Spruyt *et al.* (64)
Studies That Modeled a Generalization Gradient	
Gaussian (Tuning) Models	Dou *et al.* (65), Grosso *et al.* (66), Huang *et al.* (67), Kampermann *et al.* (68), Kausche *et al.* (69), Kausche *et al.* (70), Kausche *et al.* (71), Onat *et al.* (33), Porter *et al.* (72), Resnik *et al.* (35), Tuominen *et al.* (34), Tuominen *et al.* (73), Wang *et al.* (74), Yu *et al.* (75), Zaman *et al.* (76), Zaman *et al.* (77), Zaman *et al.* (78), Zaman *et al.* (79), Zenses *et al.* (80)
Quadratic-Linear Models	Cha *et al.* (81), Cha *et al.* (37), Cha *et al.* (82), Dunning *et al.* (38), Dymond *et al.* (83), El-Bar *et al.* (84), Hammell *et al.* (85), Laufer *et al.* (86), Wickens *et al.* (87), Zaman *et al.* (88), Zaman *et al.* (77)
Studies That Examined the Amount of Generalization at the Group Level	
ANOVA-Based Quadratic and Linear Trend	Ahmed *et al.* (89), Cooper *et al.* (40), Davidson *et al.* (90), Dunning *et al.* (38), Dunsmoor *et al.* (91), Dunsmoor *et al.* (92), Dowd *et al.* (93), Gao *et al.* (94), Glenn *et al.* (95), Glenn *et al.* (96), Greenberg *et al.* (21), Hunt *et al.* (97), Klein *et al.* (98), Klein *et al.* (99), Lange *et al.* (58), Lange *et al.* (100), Lee *et al.* (101), Li *et al.* (102), Lissek *et al.* (14), Lissek *et al.* (19), Lissek *et al.* (103), Manbeck *et al.* (104), Michalska *et al.* (105), Niederstrasser *et al.* (106), Phillips (107), Reinhard *et al.* (108), Roesmann *et al.* (109), Struyf *et al.* (110), Torrents-Rodas *et al.* (111), Vandael *et al.* (112), Vandael *et al.* (113), Van Meurs *et al.* (114), Vervliet *et al.* (30), Wong *et al.* (115), Wong *et al.* (116), Wong *et al.* (117), Wong *et al.* (118), Wong *et al.* (119), Zoladz *et al.* (120)
Quadratic and (Difference of) Gaussian Contrast Comparisons	Antov *et al.* (121), Friedl *et al.* (122), McTeague *et al.* (123), Plog *et al.* (124), Stegmann *et al.* (125)
Hierarchical Models Including Quadratic Terms	Ginat-Frohlich *et al.* (126), Ginat-Frohlich *et al.* (127), Keefe *et al.* (128), Lommen *et al.* (129), Meulders *et al.* (130)
Various Types of Regression Models	Dos Santos *et al.* (131), Fan *et al.* (132), Gao *et al.* (133), Michalska *et al.* (105), Nelson *et al.* (134), Resnik *et al.* (35), Schroijen *et al.* (135), Struyf *et al.* (36), Yu *et al.* (136), Zaman *et al.* (137)

ANOVA, analysis of variance; GI, generalization index; LDS, linear deviation score.

### Simulation Analysis: Psychometric Properties of Fear Generalization Indices

**Figure 2:**
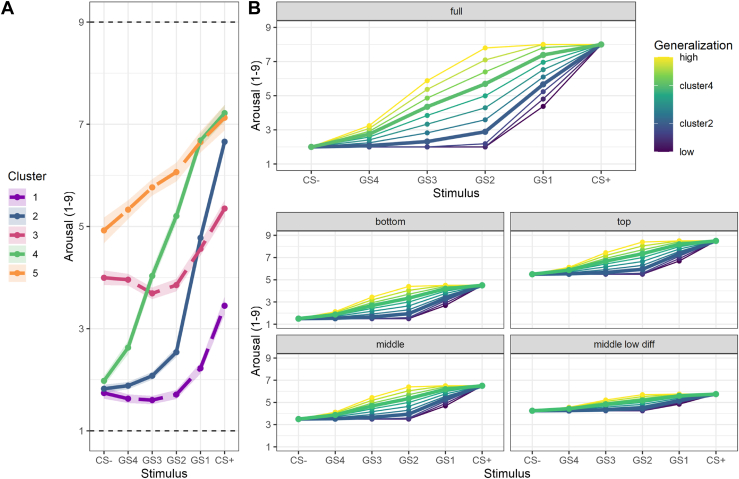
Illustration of representative fear generalization gradients spanning the continuum between conditioned stimuli (CS− and CS+). **(A)** shows the empirical basis taken from Stegmann *et al.* (27) that was used for generating the profiles illustrated in **(B)**. Clusters 2 and 4 [solid lines in **(A)**] demonstrated high differentiation between the CS+ and CS−, with cluster 4 (green) showing an earlier and more steady increase in subjective arousal from the CS− across the generalization stimuli (GS) to the CS+, while cluster 2 (blue) was characterized by a slower but then sudden increase. Therefore, clusters 2 and 4 were used as a basis for response profiles with different amounts of generalization [see the bold lines in **(B)**]. Clusters 1, 3, and 5 [dashed lines in **(A)**], on the other hand, exhibit reduced differentiation between CS+ and CS− on different levels of general response strength and therefore were used to inform the simulated profiles on a lower, middle, and high response level **(B)**. We then normalized the values of clusters 2 and 4 from 0 to 1 and created 9 additional generalization levels between as well as above and below both generalization profiles (3 interpolation steps and 3 equidistant extrapolations on either side). The resulting 11 fear generalization profiles were winsorized to assure that no GS had values more extreme than the CSs (55). The final gradients served as prototypes for 5 model cases that were tested in our simulation. The first case (full) spanned almost the full scale ranging from 2 to 8, thus showing high differentiation and an intermediate average level. We added a bottom (1.5–4.5), middle (3.5–6.5), and top (5.5–8.5) case with intermediate differentiation but different proneness to floor and ceiling effects. Lastly, we included another middle cluster (middle low diff; 4.25–5.75) similar to empirical cluster 3 but with even lower differentiation. The resulting model cases, each including 11 fear generalization profiles, thus exhibited different amounts of CS differentiation and general arousal level.

In the next step, we conducted an empirically informed simulation analysis to better understand how measurement errors influence different indices of fear generalization. To simulate representative profiles of fear generalization gradients, we relied on our previous study (27) that identified 5 clusters of profiles. These clusters were based on arousal ratings obtained for the CS+, CS−, and 4 GS on a scale from 1 (calm) to 9 (intense) in a large group of participants (*N* = 1175). The simulation analysis was based on the empirically informed cases (Figure 2) as true scores. For each of the 55 resulting gradients (5 model cases × 11 gradients per case) (see Figure 2B), we calculated the LDS and GI and extracted the curvature parameters from either a Gaussian model fit (σ_Gauss_) or an exponential model fit (λ_exp_) as well as the relative importance of the linear over the quadratic component from a quadratic polynomial model fit (%_linquad_).

To assess the psychometric properties of these indices, we then calculated their intercorrelations and examined how different indices responded to variations in the range of responses or scale restrictions across the generalization gradient. Finally, we determined the sensitivity of indices to measurement noise by determining the correlation of noise-corrupted generalization profiles from the same true scores and by assessing systematic bias of generalization indices in the presence of noise (for further details on these calculations, see the Supplement).

### Empirical Analysis: Reliability and Validity of Fear Generalization Indices

As a final step, we examined the split-half reliability and concurrent validity of the previously identified and characterized generalization indices using 2 large samples of healthy participants (*N*_1_ = 1175 and *N*_2_ = 256) (details about the samples and the experimental protocol are reported in the Supplement). Similar to the simulation analysis, we obtained the different indices of fear generalization for each participant and dependent measure, including the LDS, GI, σ_Gauss_, λ_exp_, and %_linquad_. In addition, we calculated the mean response levels and the difference between CS+ and CS− responses (CS differentiation) as basic indices of threat responsiveness for each participant, which were previously found to correlate with individual differences in anxiety (27). We also extracted the scale parameter *b*_Gauss_ and vertical translation parameter *a*_Gauss_ from the Gaussian model fit, the scale parameter *n*_exp_ and vertical translation parameter *a*_exp_ from the exponential model fit, and the intercept parameter *c*_linquad_ from the quadratic polynomial model fit. Descriptive statistics for the fear generalization indices and model parameters for subjective (arousal, unpleasantness, and US expectancy ratings) and autonomic (skin conductance) responses can be found in Table S4. Then, we conducted the following analyses: First, we calculated bivariate linear correlations between indices using Pearson’s correlation coefficients (*r*s) to examine to what degree individual measures captured the same construct. Second, to assess split-half reliability, we calculated correlations between the first and second block of the generalization phase (see the Supplement). Third, to assess concurrent validity, we correlated fear generalization indices for the whole generalization phase with questionnaire scores assessing different constructs related to anxiety, trauma, and negative affect. All statistical analyses were conducted with R version 4.1.2 (28). The alpha level was set to 0.05. All analyses were carried out separately for arousal, unpleasantness, and US expectancy ratings as well as for skin conductance responses (SCRs). Due to their similarity with arousal ratings, results for unpleasantness and US expectancy ratings are reported in the Supplement.

## Results

### Simulation Analysis: Psychometric Properties of Fear Generalization Indices

#### Correlations Between Fear Generalization Indices

**Figure 3:**
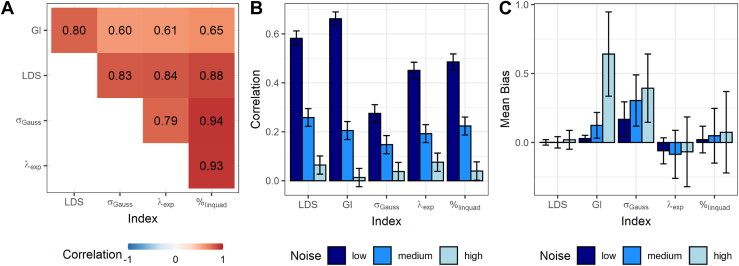
Summary of the psychometric properties of fear generalization indices. **(A)** Correlations between the individual fear generalization indices across the 55 different true-score generalization gradients (without noise). Please note that the linear deviation score (LDS) and the exponential model fit (λ_exp_) are inverted for better comparability. **(B)** Correlations between pairs of generalization profiles drawn from the same true scores at different levels of noise [medium levels reflect the empirical variability found between repeated measures in the large sample of Stegmann *et al.* (27)]. Values can be interpreted as reliability estimates. Error bars indicate 95% CIs of correlation parameter estimates. **(C)** Mean and SD of differences between true scores and estimates of generalization indices, separated by levels of noise. GI, generalization index; σ_Gauss_, Gaussian model fit; %_linquad_, quadratic polynomial model fit.

To quantify the extent to which the indices reflect the same construct, we calculated intercorrelations between them. Correlations ranged from *r* = 0.60 to 0.94, indicating a relatively strong similarity between indices (see Figure 3A). At the same time, the magnitude of correlations suggests that the indices are not identical and therefore should not be used interchangeably. On average, the strongest correlation with the other fear generalization indices was shown by the %_linquad_ (average *r* [*r*_avg_] = 0.85), closely followed by the LDS (*r*_avg_ = 0.84), the λ_exp_ (*r*_avg_ = 0.79) and the σ_Gauss_ (*r*_avg_ = 0.79). Only the GI showed substantially weaker correlations with the other indices (*r*_avg_ = 0.67). Importantly, these correlations were based on true scores and therefore can be interpreted as an upper limit for their similarity in noise-free measurements.

#### Differentiation

**Figure 4:**
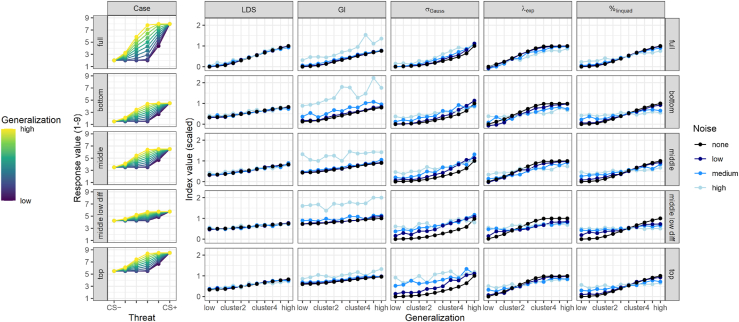
Differentiation graphs of various generalization indices separated by model case and noise level. Columns depict generalization indices for each model case (rows, depicted in first column) and noise level (blue shades). True scores without noise are depicted in black. Conformity between black and other lines indicates low bias. Higher average slopes reflect better differentiation between different generalization profiles within the same case. Susceptibility to noise is reflected by systematic deviation of the blue lines from the black line and unsystematic variance within blue differentiation graphs across the 50 pairs drawn from the same true scores. The latter is depicted in Figure S2. CS, conditioned stimuli; GI, generalization index; LDS, linear deviation score; λ_exp_, exponential model fit; σ_Gauss_, Gaussian model fit; %_linquad_, quadratic polynomial model fit.

Next, we examined how different indices respond to variations in the response range and scale restrictions across the generalization gradient. Both LDS and GI are sensitive to variations in the range of responses and show less differentiation between generalization profiles with reduced response ranges (LDS: 100%, 50%, and 25%; GI: 76%, 47%, and 26% differentiation for full, middle, and low differentiation cases, respectively) (cf. Table S2). Thus, LDS and GI interpret the profiles of the full model case as more diverse than other cases even though the latter are simply scaled variants of the full model case. The GI factors in the absolute level of the CS+ rendering profiles as less diverse that are shifted upwards (bottom: 67%, middle: 47%, top: 36% differentiation). The model-fitted indices showed no such dependency on the variance of the responses (differentiation ≥98%). However, their superior differentiation is merely theoretical. As soon as noise was included and true scores were not accessible, their differentiation decreased similarly to that of the LDS or GI (Figure 4).

#### Robustness Against Noise

To assess the sensitivity of generalization indices to measurement noise, we introduced 3 levels of random noise and correlated the resulting indices with their corresponding true scores as an indicator of robustness. Correlations between pairs of generalization profiles drawn from the same true scores at different levels of noise indicated that the LDS showed the greatest robustness against noise. Only the GI showed a higher correlation at low noise, but robustness quickly decreased at medium noise and was lowest of all indices for high noise. Exponential and quadratic fits showed an overall similar pattern of susceptibility to noise, while Gaussian fits did not perform well across all noise levels (Figure 3B). While the LDS exhibits low bias even at high levels of noise, the GI is highly susceptible to increasing noise. Gaussian fits exhibited bias of overestimation already at low levels of noise (Figure 3C). Exponential and quadratic polynomial fits have no overall bias across different generalization profiles and model cases but show systematic deviations from the true scores for certain combinations of variables. Specifically, quadratic polynomial fits have a tendency toward a medium generalization estimate, and exponential fits show a similar tendency, but their bias is shifted into the direction of cluster 2 (low generalization), thus representing a significant underestimation of generalization strength (Figure 4).

### Empirical Analysis: Reliability and Validity of Fear Generalization Indices

#### Correlations of Fear Generalization Indices

**Figure 5:**
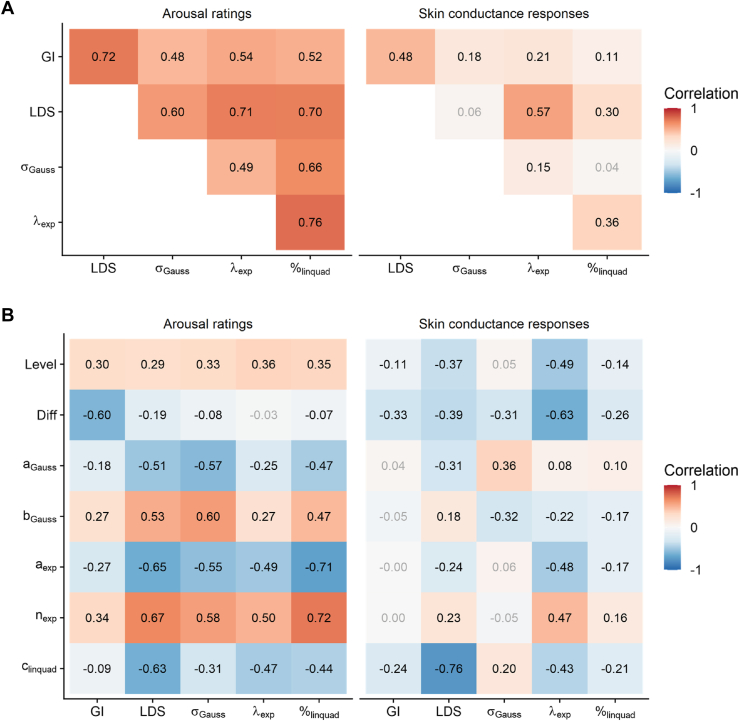
Correlations between different indices of fear generalization and their correlations with basic indices of threat responsiveness. Pearson’s correlations between different indices of fear generalization are depicted in **(A)**, while **(B)** depicts their correlations with basic indices of threat responsiveness. Statistically significant correlations are printed in black. Please note that the linear deviation score (LDS) and the exponential model fit (λ_exp_) are inverted for better comparability. % indicates relative importance of the linear compared to the quadratic term. CS, conditioned stimulus; Diff, CS differentiation; Gauss, Gaussian model fit; GI, generalization index; Level, mean response level; linquad, quadratic polynomial model fit.

Intercorrelations between indices of fear generalization were generally higher for subjective arousal than for SCR amplitudes. Consistent with the simulation analyses, the pattern of correlations indicates that the different indices tap into the same construct but are not identical and should not be used interchangeably (see Figure 5A). The correlational analysis between generalization measures and basic indices of threat responsiveness (see Figure 5B) revealed generally moderate associations, suggesting that none of the curvature parameters can be adequately explained by mean response levels or CS differentiation. Furthermore, consistent negative correlations were observed between fear generalization indices and their respective vertical translation as well as intercept parameters (*a*_Gauss_, *a*_exp_, and *c*_linquad_), while positive correlations were found with their respective scaling parameters (*b*_Gauss_ and *n*_exp_). These results were less consistent for SCR amplitudes and highly stable across both samples (see Figures S7 and S8).

#### Split-Half Reliability

**Figure 6:**
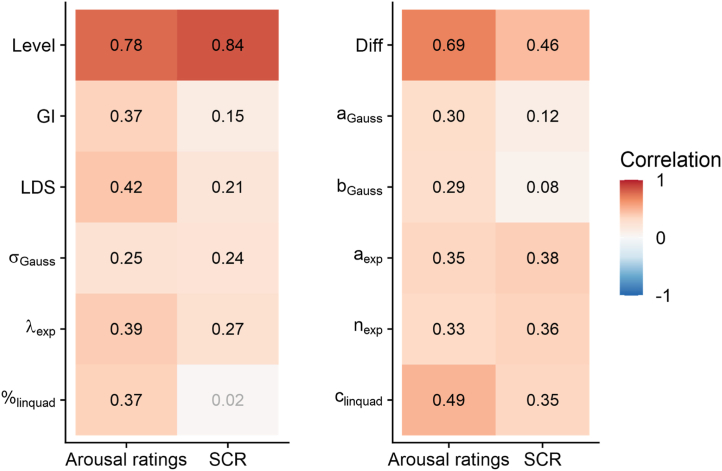
Correlations between measures of threat responsiveness and generalization of the first and second half of the fear generalization phase. % indicates relative importance of the linear compared to the quadratic term. Please note that the linear deviation score (LDS) and the exponential model fit (λ_exp_) are inverted for better comparability. CS, conditioned stimulus; Diff, CS differentiation; exp, exponential model fit; Gauss, Gaussian model fit; GI, generalization index; Level, mean response level; linquad, quadratic polynomial model fit; SCR, skin conductance response.

Correlations between the first and the second generalization block were generally higher for arousal ratings than for SCRs (see Figure 6), with the highest reliabilities for mean response levels (arousal: *r* = 0.78, SCR: *r* = 0.84) and CS differentiation (arousal ratings: *r* = 0.69, SCR amplitudes: *r* = 0.46), while reliability scores for the different indices of fear generalization were substantially lower (arousal ratings ranged from *r* = 0.25 to 0.49, SCR amplitudes ranged from *r* = 0.02 to 0.38). Again, we found no systematic differences between different indices of gradient curvature.

#### Concurrent Validity

**Figure 7:**
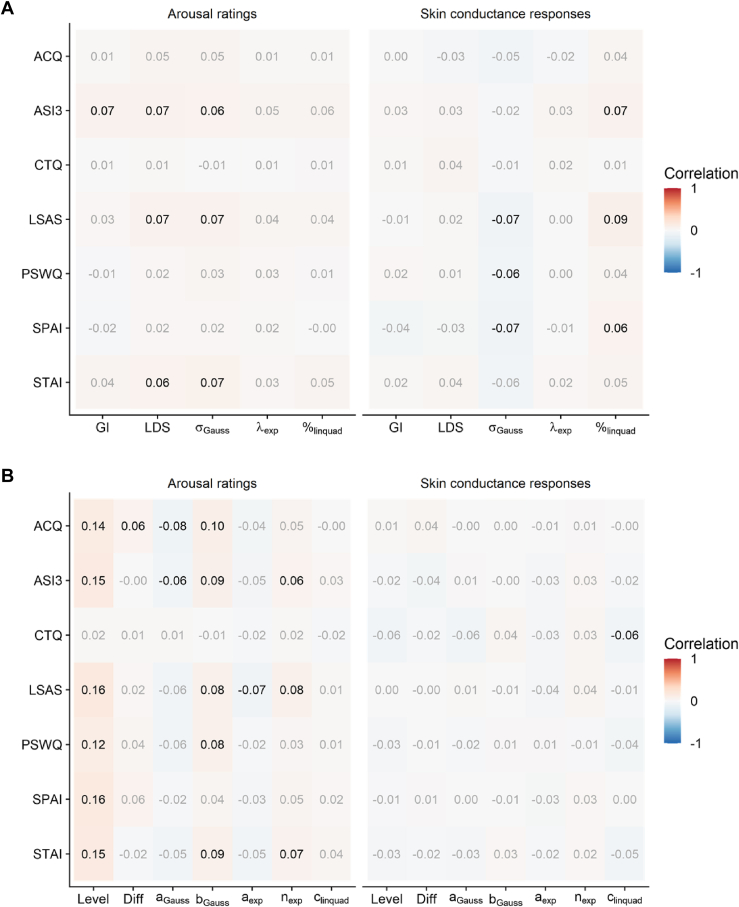
Correlations between questionnaire measures of anxiety and indices of fear generalization and general threat responsiveness. Correlations of indices of fear generalization **(A)** and basic indices of threat responsiveness **(B)** with various measures of anxiety and related constructs. % indicates relative importance of the linear compared to the quadratic term. Please note that the linear deviation score (LDS) and the exponential model fit (λ_exp_) are inverted for better comparability. ACQ, Agoraphobic Cognition Questionnaire; ASI3, Anxiety Sensitivity Index 3; CS, conditioned stimulus; CTQ, Childhood Trauma Questionnaire; Diff, CS differentiation; exp, exponential model fit; Gauss, Gaussian model fit; GI, generalization index; Level, mean response level; linquad, quadratic polynomial model fit; LSAS, Liebowitz Social Anxiety Scale; PSWQ, Penn State Worry Questionnaire; SPAI, Social Phobia Anxiety Index; STAI-T, State-Trait Anxiety Inventory-Trait.

Because overgeneralization of learned fear is frequently discussed as a key mechanism contributing to the etiology and maintenance of anxiety disorders (20,21), we compared the different fear generalization indices regarding their performance in predicting individual differences in anxiety and related constructs as measured by questionnaires. In the first sample (*N*_1_ = 1175), correlations were generally weak, and we were unable to identify any systematic relationship between questionnaire measures and fear generalization indices. This lack of correlation was evident across ratings and SCRs (see Figure 7A). In the second sample (*N*_2_ = 256), we replicated these results, although we found slightly stronger associations for arousal ratings (see Figure S9). Interestingly, analyses of the basic indices of threat responsiveness revealed slightly higher correlations (see Figure 7B). For arousal ratings, but not for SCRs, we identified systematic associations between mean response levels and measures of anxiety in both samples (see Figure S10).

## Discussion

In the current article, our aims were 3-fold: first, to identify commonly used methods for characterizing fear generalization profiles in the literature; second, to describe the psychometric properties of these indices, their robustness against measurement noise, and their interrelations using simulations; and third, to examine the split-half reliability and concurrent validity of these different approaches for quantifying fear generalization on the individual level, we used 2 large datasets (*N* = 1175 and *N* = 256).

The literature review revealed large heterogeneity in the quantification of individual fear generalization profiles. While most studies focused on group-level effects [e.g., (19,29,30)], those that examined interindividual differences reported considerable variability in the indices used. The most frequently used measures included the LDS (14,31), GI (32), and curvature parameters obtained from model-fitting approaches based on Gaussian functions [e.g., (33,34)], exponential functions (35,36), or quadratic polynomials (37,38). All these indices were based on theoretical considerations or were justified based on empirically observed fear generalization profiles. None of the indices provides a normalized outcome as their ranges depend on the measured variable (and in the case of the GI, also on the number of GS), which makes it difficult to compare results between studies.

To gain insight into the psychometric properties of different indices, we conducted a simulation analysis, systematically examining the interrelation of indices and their robustness against measurement noise. Comparing the individual fear generalization indices across the simulated gradients revealed generally strong correlations up to *r* = 0.94, suggesting that all indices tap into the same construct. Crucially, these scores are a theoretical upper limit for similarity, while we assume lower correlations for more noisy measurements. Consistent with this assumption, analyses of empirical data revealed moderate to high correlations (0.48 ≤ *r* ≤ 0.76) for arousal ratings but substantially lower values for SCR amplitudes (0.04 ≤ *r* ≤ 0.57). These findings have several important implications. While each index can in principle be used to describe the curvature of individual generalization gradients, they are not equivalent and should not be used interchangeably. However, based on the intercorrelations alone, we were unable to identify a preferable index, which suggests that other factors should be considered when choosing a suitable generalization index.

One of these factors is the robustness against unsystematic noise, which can be particularly relevant for clinical studies. Simulations across 3 different levels of noise revealed that all generalization indices exhibited unexpectedly low robustness even at moderate noise levels. These findings are supported by the split-half reliabilities of the empirical data, which were in fact moderate for arousal ratings (0.25 ≤ *r* ≤ 0.42) but strikingly low for SCR amplitudes (0.02 ≤ *r* ≤ 0.27). Thus, generalization indices do not seem reliable under conditions of high measurement noise. This is not surprising as previous studies also found modest reliabilities in fear generalization paradigms (39,40). Therefore, monitoring and eliminating any potential sources of measurement noise in fear generalization research is advised (26).

While the previously discussed issues apply to all indices, our simulation analysis also revealed crucial differences between them. The LDS was the most robust index against noise and exhibited minimal bias. Nevertheless, the LDS showed less differentiation between generalization profiles when the response range was restricted. This could reduce between-subjects variance and thus affect reliability and interindividual differences measures (26). In contrast, model-fitted indices did not depend on the response range, but we detected distinct differences among them. The σ_Gauss_ overestimated the generalization curvature, especially in conditions with elevated noise. The exponential (λ_exp_) and quadratic (%_linquad_) fits showed some robustness for highly noisy data but also demonstrated stronger bias than the LDS. Compared with the other indices, the GI presented several negative features: It was the most sensitive index to varying levels of noise and overestimated generalization under high noise. Similar to the LDS, the GI did not fully differentiate between generalization profiles if the response range was restricted. These problematic issues can be attributed to 2 factors. First, the GI excludes responses to CS−, and second, it emphasizes the response to CS+ as it is the only variable in the denominator, which makes it particularly susceptible to measurement noise.

Having observed these differences between fear generalization indices, we assume that these aspects affect how well the indices capture individual differences in anxiety. In a recent study, participants who exhibited the broadest fear generalization (i.e., highest LDS scores) also demonstrated elevated levels of trait anxiety, although the effect sizes were small (27). Here, we reanalyzed these data by focusing on individual generalization profiles instead of group effects. Correlational analyses revealed no systematically significant associations between different indices and questionnaire measures of individual anxiety and related traits. In addition, no differences were found between the indices, indicating that they all performed relatively poorly. To substantiate these findings, all analyses were repeated in an independent sample. Even though individual correlations were slightly stronger, the overall pattern of results remained similar, showing no systematic associations between measures of fear generalization and questionnaire scores. Previous studies suggested that threat responsiveness in fear generalization paradigms should not only be characterized by the curvature of generalization gradients but should also account for more basic measures such as the general response strength and the differentiation between CS+ and CS− (27,41). While these additional measures are complementary to the LDS and GI, model-fitting approaches capture these characteristics with their remaining free parameters, e.g., the vertical translation and scaling factors of the Gaussian as well as the exponential functions or the intercept parameter of the quadratic polynomial. However, these basic measures of threat responsiveness did not perform any better in predicting individual differences in anxiety, except for mean response levels showing small but consistent correlations with most questionnaire scores in both samples. This is consistent with previous observations that mean response levels appear to be a better predictor of individual differences than the curvature of the fear generalization gradient (27). An explanation for these relatively weak correlations can be derived from our simulation and empirical analyses, which demonstrated low reliabilities for all fear generalization indices. From a theoretical perspective, these reliabilities provide the upper limit for correlations with individual difference measures (26). This also explains the relatively consistent correlations between questionnaire measures and the more reliable mean response levels.

Collectively, the current findings raise the question of how the phenomenon of fear generalization can be adequately conceptualized and quantified. Researchers have predominantly relied on indices that describe the curvature (or deviation from linearity) of the fear generalization gradient. However, because fear generalization indices are not independent of basic parameters such as mean response levels or CS differentiation, this narrow conceptualization of fear generalization may not be sufficient for future research. Therefore, we propose that threat responsiveness should be characterized more comprehensively by quantifying fear generalization gradients together with mean response levels and the differentiation between CS+ and CS−. Alternatively, model-fitting approaches can overcome this problem with their additional free parameters. Consistent with this, future research may benefit from incorporating multivariate analytical approaches to better capture the complex interplay between different generalization indices and basic parameters of threat responsiveness. While the current study relied on standard practices in the field and focused on evaluating individual indices separately, such multivariate methods represent a promising approach for advancing the conceptual and methodological framework of fear generalization research.

Although the current study provides a broad overview and extensive assessment of fear generalization measures, it is important to note that our analyses relied on one standard preprocessing pipeline for all empirical data. However, our simulation suggests that some indices of fear generalization are sensitive to transformations of the response variables. The field of fear conditioning research has begun to address the multitude of possible transformations with the help of multiverse or specification curve analyses (42, 43, 44, 45) that consider all plausible preprocessing/analysis combinations, thus providing a detailed comparison of the different methodologies (46). Consistent with our findings, recent studies have demonstrated inconsistencies in the use of acquisition and extinction measurements (47,48), which persist regardless of variations in sample sizes (49). Further supporting our results, recent findings showed that only a small subset of fear conditioning analysis pipelines provided measures that correlated with anxiety traits (50). Similarly, it is important to note that all empirical analyses of the current study were based on a fear generalization task using facial stimuli, whereas other paradigms often use simpler cues, such as geometric shapes that vary in size (19). This may limit the generalizability of our empirical findings to some extent, but we aimed to account for this with our simulation approach. For example, stimulus material that is more prone to perceptual errors may impair fear discrimination across the stimulus range and increase measurement noise (51), two relevant factors that were explicitly addressed in the simulation analysis. Another limitation is that the current analyses relied on relatively homogeneous samples of young and healthy participants, while including more diverse participants and patients with anxiety might have been beneficial for increasing reliability estimates. In fact, however, we observed substantial variability in the questionnaire scores and the fear generalization measures, which is consistent with the conceptualization of psychopathology as a continuum (52), but we failed to observe a relationship between the individual degree of fear generalization and anxiety-related traits. Therefore, it seems relevant to replicate the current analyses in patient groups. Finally, it is important to note that the current approaches to quantifying fear generalization are limited to perceptual generalization, as they rely on varying GS along a continuum of similarity to the CS+ or CS−. However, other forms of fear generalization, e.g., conceptual generalization (53,54), may be equally relevant for understanding individual differences in anxiety and for investigating their role in the etiology and maintenance of anxiety disorders, although they may require distinct analytical approaches.

### Conclusions

We identified substantial heterogeneity in the literature on how individual fear generalization profiles can be quantified. However, we were able to identify a few common indices including the LDS, GI, and parameters extracted from model-fitting approaches. Exploring their robustness against measurement noise, their split-half reliability, and their validity in predicting individual differences in anxiety, we demonstrated large conceptual overlap among indices but also substantial sensitivity to noise. None of the indices predicted anxiety and related constructs well, whereas mean response levels, as a more general index of threat responsiveness, were more robustly associated with trait anxiety. Taken together, the current results highlight the need for more systematic methodological research to reduce the heterogeneity in the literature and enable informed decisions on how to reliably estimate fear generalization from subjective and physiological data. We propose to consider basic aspects of threat responsiveness alongside fear generalization indices to characterize individual differences in threat processing more comprehensively, thereby improving future research on risk factors for the etiology and maintenance of anxiety disorders. Finally, the current approach consisting of a comprehensive literature review, empirically informed simulation analyses, and examinations of empirical data from large samples could also serve as a blueprint for further studies on psychological mechanisms that contribute to the emergence of psychopathology.

## Supplementary Material

Supplemental Methods, Results, Figures S1–S10, and Tables S1–S4

Key Resources Table
