# Supplementary material for: Psychometric Considerations in Assessing Fear Generalization as a Predictor of Anxiety
Source: Biol Psychiatry Glob Open Sci. 2025 Jul 25;5(6):100570. doi: 10.1016/j.bpsgos.2025.100570 (PMC12454897; doi:10.1016/j.bpsgos.2025.100570)
Supplement: Supplemental Methods, Results, Figures S1–S10, and Tables S1–S4 [file mmc1.pdf]

SUPPLEMENTARY INFORMATION

Psychometric Considerations in Assessing Fear Generalization as a Predictor of Anxiety

Stegmann *et al.*

Content

Literature Review ... 3
  Article Search ... 3
  Description of Indices ... 4
Simulation analysis ... 17
Empirical analysis ... 20
  Methods ... 20
    Samples ... 20
    Experimental protocol ... 21
    Physiological data processing ... 22
  Results ... 23

Figure S1. PRISMA Flowchart of Study Selection and Inclusion ... 3
Figure S2. Unsystematic Variance between Pairs of Gradients drawn from the Same True Scores for Various Generalization Indices Separated by Model Case and Noise Level ... 19
Figure S3. Correlations Between Different Indices of Fear Generalization (Panel A) and their Correlations with Basic Indices of Threat Responsiveness (Panel B) for Unpleasantness and US Expectancy Ratings. . 25
Figure S4. Correlations Between Various Parameters of the First and Second Half of the Fear Generalization Phase for Unpleasantness and US Expectancy Ratings ... 26
Figure S5. Correlations Between Trait Measures of Anxiety Psychopathology and Indices of Fear Generalization (Panel A) as well as Basic Indices of Fear Responsiveness (Panel B) for Unpleasantness and US Expectancy Ratings ... 27
Figure S6. Comparison of Correlations Among Gradient Curvature Parameters Between the First Sample and the Second Sample ... 27
Figure S7. Comparison of Correlations Among Gradient Curvature Parameters and Basic Parameters of Fear Generalization Between the First Sample and the Second Sample ... 28
Figure S8. Comparison of Correlations Among Gradient Curvature Parameters and Trait Measures of Anxiety Psychopathology Between the First Sample and the Second Sample. ... 29
Figure S9. Comparison of Correlations Among Basic Parameters and Trait Measures of Anxiety Psychopathology Between the First Sample and the Second Sample ... 30
Figure S10. Comparison of Correlations Among Basic Parameters and Individual Differences in Anxiety-Related Constructs Between the First Sample and the Second Sample ... 31

Table S1. Overview of the Studies for the Different Experimental Settings ... 9
Table S2. Normalized Range Covered by Each Generalization Index per Model Case ... 18
Table S3. Summary of the Sample Characteristics ... 20
Table S4. Summary of Generalization Indices and Model Parameters of Study 1 (n = 1175) and Study 2 (n = 256) ... 23

Literature Review

Article Search

In order to identify commonly used generalization indices, we conducted a systematic, non-pre-registered literature review closely following the PRISMA guidelines [1, 2]. The search was carried out in Web of Science and in PsycInfo. Articles published between 1990 and (1st November) 2024 as well as written in English were considered. Reviews, conference abstracts, books, and editorials were excluded. The terms for the search included: “fear generalization” OR “generalization gradient” OR “generalization processes” OR “memory generalization”.

Figure S1. *PRISMA Flowchart of Study Selection and Inclusion*

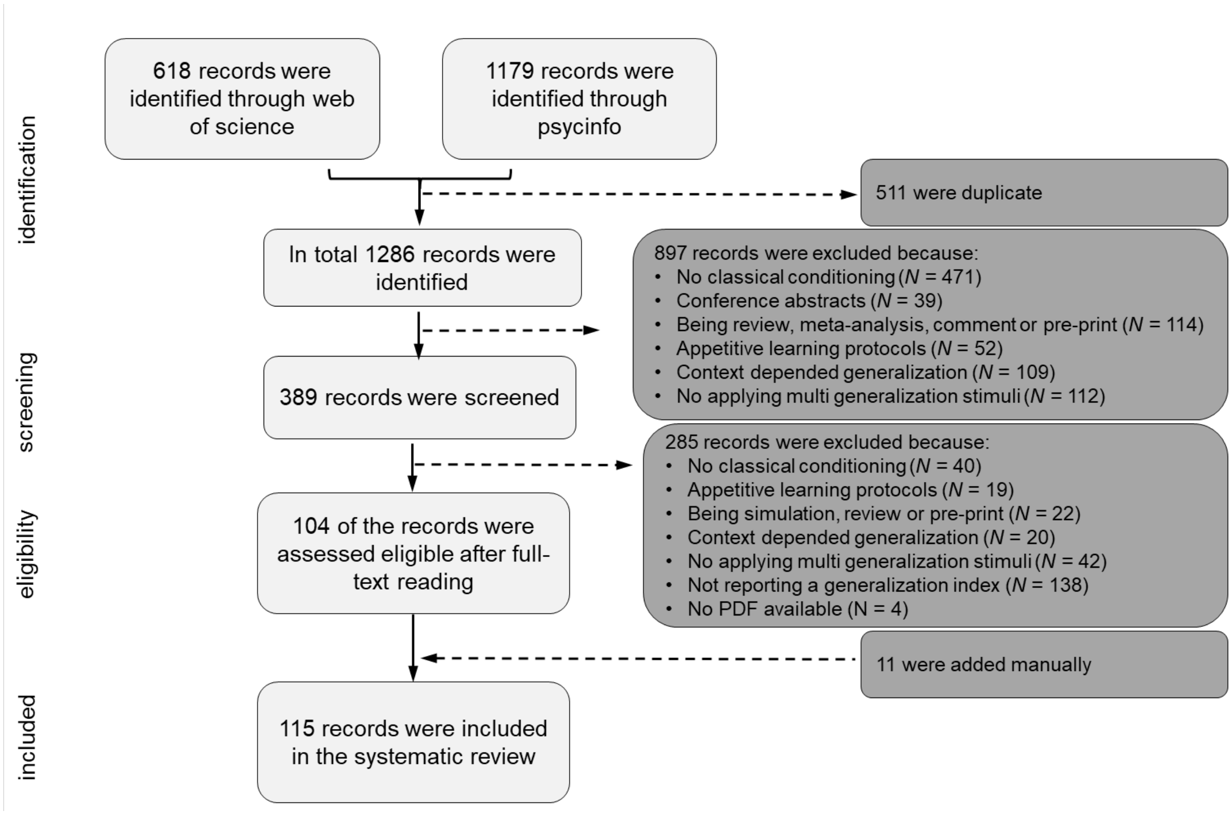

The search returned 618 articles from the Web of Science and 1179 articles from PsycInfo, with 511 being duplicates. Articles were then screened based on their abstract. In order to be considered for

this systematic review, the studies had to meet the following criteria: 1) being a classical conditioning study; 2) not being a conference abstract; 3) not being a review, comment or meta-analysis; 4) not relying on appetitive classical conditioning with a rewarding unconditioned stimulus (US); 5) not using a context-dependent generalization design (i.e., conditioned defensive responses are generalized according to context rather than stimulus similarity); and 6) generalization of conditioned defensive responses was tested over multiple stimuli. The eligible articles were 389, which were read and evaluated in detail focusing on the above-mentioned criteria. After a careful evaluation, 104 records were considered for the systematic review, and eleven articles were manually identified and added (see Figure S1).

Description of Indices

Arithmetical indices

The most commonly used arithmetic index to describe the curvature of the generalization gradient is the Linear Deviation Score [LDS, 3], which can be calculated as

$$LDS = \frac{CS^{+} + CS^{-}}{2} - \frac{GS_1 + GS_2 + GS_3 + \cdots + GS_n}{n}$$

where CS^{+} is the individual response to the conditioned stimulus that was followed by the US (CS+), CS^{-} is the response to the stimulus signaling safety (CS−) and GS_n is the response to the nth level of the generalization stimuli (GS). The LDS can be illustrated as a measure of how strongly the responses to the GSs deviate from a hypothetical straight line between CS− and CS+ responses. Values near zero indicate a linear gradient, whereas negative values indicate stronger generalization, respectively (for an illustration of LDS values for different generalization profiles, see Figure 1A). Please note that we changed the polarity of the LDS (i.e., higher values indicate stronger generalization) for the analyses in order to

achieve better comparability with other fear generalization indices. The range of the LDS is not restricted and varies depending on the range and the units of the measured variables.

A second frequently used method to describe the curvature of fear generalization gradients is the generalization index [GI, 4], which is calculated by dividing the sum of the responses to the GSs by the response to the CS+:

$$GI = \frac{GS_1 + GS_2 + GS_3 + \cdots + GS_n}{CS^+}$$

The GI can be considered as the combined response strength to the GS relative to the original fear stimulus (see Figure 1A). When interpreting the values of the GI, it is crucial to consider the number of generalization stimuli and the numerical range of the measured variable. Higher values indicate stronger fear generalization. In addition, it is important to note that (1) the GI does not include responses to the CS− and (2) the calculation of the GI will result in missing values if responses to the CS+ are zero (as for example sometimes observed for skin conductance response amplitudes).

Model fitting approaches

Apart from indices that rely on an arithmetic combination of responses, several studies employed model fitting approaches that describe the individual fear generalization gradient by statistically fitting a pre-defined function and extracting its parameters. The choice of function was based either on assumed theoretical considerations or subjective preferences. To determine the best fit between the model function and the generalization gradient, least squares are often used that involve finding the values of the model parameters that minimize the sum of the squared differences between the predicted and the observed data. The residuals can also be used to calculate goodness of fit parameters (e.g., R^2), which provide an estimate of how well the model fits the generalization profile.

The most frequently used function in the literature is the Gaussian function [Gaussian model fit, Gauss; e.g. 5, 6], which can be expressed as

$$y = a + b * (\frac{1}{\sigma\sqrt{2\pi}} e^{-\frac{1}{2}\left(\frac{x-n+2}{\sigma}\right)^2})$$

with

$$y = \begin{pmatrix} CS^- \\ GS_1 \\ \dots \\ GS_n \\ CS^+ \\ GS_n \\ \dots \\ GS_1 \\ CS^- \end{pmatrix} and\ x = \begin{pmatrix} 1 \\ 2 \\ \dots \\ n+1 \\ n+2 \\ n+3 \\ \dots \\ 2n+2 \\ 2n+3 \end{pmatrix}$$

CS^+ is the individual response to the CS+, CS^- is the response to the CS− and GS_n is the response to the nth level of the generalization stimuli. The Gaussian function has three free parameters: the standard deviation of the normal distribution σ_{Gauss}, the scale factor b_{Gauss}, and the vertical translation factor a_{Gauss}. Assuming the other parameters remain constant, increases in σ_{Gauss} widen the “bell”-shape of the Gaussian function, increases in b_{Gauss} increase the maximum level at CS+, and increases in a_{Gauss} increase the baseline of the Gaussian. Thus, σ_{Gauss} is the best single parameter to describe the curvature of the generalization gradient, with higher values indicating more generalization. The Gaussian model suits naturally as description for fear generalization gradients where the CS+ lies in the center of the continuum but can also be used for gradients that extend from CS− to CS+. In these cases, the generalization gradient is conceptualized as the half of a normal distribution. Consequently, individual data needs to be mirrored at the CS+ to obtain a full normal distribution before submitting them to the fitting algorithm (see Figure 1B).

As an alternative to the Gaussian function, the exponential function has been often used to describe generalization gradients [exponential model fitting, exp; 7, 8]:

$$y = a + n * e^{\lambda x}$$

with

$$y = \begin{pmatrix} CS^- \\ GS_1 \\ GS_2 \\ \dots \\ GS_n \\ CS^+ \end{pmatrix} \; and \; x = \begin{pmatrix} 1 \\ 2 \\ 3 \\ \dots \\ n+1 \\ n+2 \end{pmatrix}$$

As fitted parameters, the algorithm returns the growth factor λ_{exp}, the scale factor n_{exp}, and the vertical translation factor a_{exp}. Assuming the other factors remain constant, changes in λ_{exp} and n_{exp} affect the steepness, while a_{exp} characterizes the horizontal asymptote of the exponential function. When both λ_{exp} and n_{exp} are positive, the exponential function is concave to the left and the horizontal asymptote represents the minimum. Conversely, if both λ_{exp} and n_{exp} are negative, the function graph is concave to the right and the horizontal asymptote represents the maximum of the exponential function. The exponential growth factor λ_{exp} is primarily responsible for the curvature of the function and therefore most accurately describes the strength of generalization. Higher values indicate reduced generalization (see Figure 1C). Similar to the LDS, we changed the polarity of the λ_{exp} (i.e., higher values indicate stronger generalization) for the analyses in order to achieve better comparability with the other fear generalization indices.

Model fitting approaches also provide an opportunity to retrieve individual fear generalization parameters analogous to the linear and quadratic trends, which are the most frequently reported for

group-level analyses, by fitting a polynomial function including a quadratic, linear, and constant term [quadratic polynomial, linquad, e.g. 9, 10]:

$$y = a + bx + cx^2$$

where

$$y = \begin{pmatrix} CS^- \\ GS_1 \\ GS_2 \\ \dots \\ GS_n \\ CS^+ \end{pmatrix} \; and \; x = \begin{pmatrix} 1 \\ 2 \\ 3 \\ \dots \\ n+1 \\ n+2 \end{pmatrix}$$

As fitted parameters, the algorithm returns the weight of the quadratic term c, the weight of the linear term b, and the intercept parameter $a_{linquad}$ (see Figure 1D). To combine the weights of the quadratic and linear terms into a single parameter, one can calculate the relative importance of the linear term ($\%_{linquad}$) over the quadratic term, which is expressed as the relative contribution of the linear term to the total explained variance [11].

Table S1. *Overview of the Studies for the Different Experimental Settings*

Reference	*Sample type*	*Learning*	*n. GSs*	*Type of US*	*Type of CS/GS*	*US-contingency*
Studies including an arithmetic index to quantify fear generalization: LDS						
Aslanidou et al. [12]	Humans (*N* = 88)	Differential cue	4	Desperate female scream	Faces	Acq: 40-80%<br>Test: 20%
Imholze et al. [13]	Humans (*N* = 441)	Differential cue	4	Desperate female scream	Faces	Acq: 83%<br>Test: 50%
Kackurkin et al. [14]	Humans (*N* = 71)	Differential cue	3	Electric stimulation	Geometrical shapes	Acq: 80%<br>Test: 50%
Lange et al. [15]	Youth (*N* = 113)	Differential cue	5	Electric stimulation	Geometrical shapes	Acq: 50%<br>Test: n.a.
Lange et al. [16]	Humans (*N* = 46)	Differential cue	5	Electric stimulation	Geometrical shapes	Acq: 66%<br>Test: 50%
Lissek et al. [14]	Humans (*N* = 48)	Differential cue	8	Electric stimulation	Geometrical shapes	Acq: 75%<br>Test: 50%
Reuter & Gamer [17]	Humans (*N* = 44)	Differential cue	4	Electric stimulation	Faces	Acq: 75%<br>Test: 50%
Stegmann et al. [18]	Humans (*N* = 1175)	Differential cue	4	Desperate female scream	Faces	Acq: 83.3%<br>Test: 50%
Zhu et al. [19]	Humans (*N* = 114)	Differential cue	3	Electric stimulation	Geometrical shapes	Acq: 80%<br>Test: 33%
Studies including an arithmetic index to quantify fear generalization: GI						
Herzog et al. [20]	Humans (*N* = 80)	Differential cue	4	Desperate female scream	Faces	Acq.: 83%<br>Test: 50%
Lenaert et al. [4]	Humans (*N* = 25)	Differential cue	6	Symbol of electricity	Faces	Acq: 75%<br>Test: 50%
Mertens et al. [21]	Humans (*N* = 120)	Differential cue	3	Electric stimulation	Words	Acq: 75%<br>Test: 50%
Reinhard et al. [22]	Children (*N* = 188)	Differential cue	4	Desperate female scream	Faces	Acq: 82%<br>Test: 50%

Spruyt et al. [23]	Humans (*N* = 88)	Differential cue	16	Aversive pictures	Faces	Acq: 100%<br>Test: n.a.
Studies modelling a generalization gradient: Gaussian (tuning) models						
Dou et al. [24]	Humans (*N* = 58)	Differential cue	6	Electric stimulation	Geometrical shapes	Acq: 75%<br>Test: 10%
Grosso et al. [25]	Rats (*N* = n.a.)	Single cue	3	Electric stimulation	Sounds	Acq: 100%<br>Test: n.a.
Huang et al. [26]	Humans (*N* = 44)	Differential cue	6	Electric stimulation	Geometrical shapes	Acq: 75%<br>Test: 50%
Kampermann et al. [27]	Humans (*N* = 74)	Differential cue	6	Electric stimulation	Faces	Acq: 30%<br>Test: n.a.
Kausche et al. [5]	Humans (*N* = 64)	Differential cue	6	Electric stimulation	Faces	Acq: 30%<br>Test: 30%
Kausche et al. [28]	Humans (*N* = 109)	Differential cue	6	Electric stimulation	Faces	Acq: 23%<br>Test: 23%
Kausche et al. [29]	Humans (*N* = 136)	Differential cue	6	Electric stimulation	Faces	Acq: 23%<br>Test: 23%
Onat & Büchel [6]	Humans (*N* = 29)	Differential cue	6	Electric stimulation	Faces	Acq: 30%<br>Test: 30%
Porter et al. [30]	Humans (*N* = 30)	Differential cue	11	Electric stimulation	Odors	Acq: 75%<br>Test: 0%
Resnik & Paz [7]	Monkey (*N* = 2)	Single cue	7	Odor	Sounds	Acq: n.a.<br>Test: n.a.
Tuominen et al. [31]	Humans (*N* = 59)	Differential cue	5	Electric stimulation	Faces	Acq: 62%<br>Test: 50%
Tuominen et al. [32]	Humans (*N* = 38)	Differential cue	5	Electric stimulation	Faces	Acq: 62.5%<br>Test: 100%
Wang et al. [33]	Humans (*N* = 62)	Differential cue	8	Aversive pictures	Geometrical shapes	Acq: 75%<br>Test: 50%
Yu et al. [34]	Humans (*N* = 80)	Differential cue	24	Electric stimulation	Geometrical shapes	Acq: 50%<br>Test: n.a.

Zaman et al. [35]	Humans (*N* = n.a.)	Differential cue	9	Electric stimulation	Colors	Acq: 75%<br>Test: n.a.
Zaman et al. [36]	Humans (*N* = 200)	Differential cue	10	Aversive pictures	Geometrical shapes	Acq: 87%<br>Test: 50%
Zaman et al. [37]	Humans (*N* = 133)	Single cue	7	Aversive pictures	Geometrical shapes	Acq: 80%<br>Test: n.a.
Zaman et al. [38]	Humans (*N* = 43)	Single cue	7	Electric stimulation	Geometrical shapes	Acq: 50%<br>Test: n.a.
Zenses et al. [39]	Humans (*N* = 40)	Single cue	7	Electric stimulation	Geometrical shapes	Acq: 80%<br>Test: 80%
Studies modelling a generalization gradient: Quadratic-linear models or means						
Cha et al. [40]	Humans (*N* = 51)	Differential cue	6	Electric stimulation	Geometrical shapes	Acq: 100%<br>Test: 50%
Cha et al. [9]	Humans (*N* = 54)	Differential cue	6	Electric stimulation	Geometrical shapes	Acq: 100%<br>Test: 50%
Cha et al. [41]	Humans (*N* = 54)	Differential cue	6	Electric stimulation	Geometrical shapes	Acq: 100%<br>Test: 50%
Dunning et al. [10]	Humans (*N* = 115)	Single cue	6	Electric stimulation	Geometrical shapes	Acq: n.a.<br>Test: 80%
Dymond et al. [42]	Humans (*N* = 50)	Differential cue	6	Aversive sound	Scenes	Acq: 67%<br>Test: 50%
El-Bar et al. [43]	Youth (*N* = 40)	Differential cue	12	Loss of points	Sounds	Acq: 100%<br>Test: 0%
Hammell et al. [44]	Humans (*N* = 52)	Differential cue	3	Electric stimulation	Geometrical shapes	Acq: 80%<br>Test: 33%
Laufer et al. [45]	Humans (*N* = 44)	Differential cue	12	Loss of money	Sounds	Acq: 100%<br>Test: 0%
Wickens et al. [46]	Humans (*N* = 144)	Single cue	2	Electric stimulation	Sounds	Acq: 100%<br>Test: 0%
Zaman et al. [47]	Humans (*N* = 105)	Differential cue	12	Symbol of electricity	Color	Acq: 75%<br>Test: 75%

Zaman et al. [48]	Humans (*N* = 200)	Differential cue	10	Aversive pictures	Geometrical shapes	Acq: 87.5%<br>Test: n.a.
Studies examining the strength of generalization on the group level: ANOVA-based linear and quadratic trends						
Ahmed et al. [49]	Humans (*N* = 59)	Differential cue	2	Electric stimulation	Geometrical shapes	Acq: 100%<br>Test: n.a.
Cooper et al. [50]	Humans (*N* = 46)	Differential cue	6	Electric stimulation	Sounds	Acq: 40%<br>Test: n.a.
Davidson et al. [51]	Humans (*N* = 33)	Differential cue	8	Electric stimulation	Geometrical shapes	Acq: 77%
Dunsmoor et al. [52]	Humans (*N* = 42)	Differential cue	6	Electric stimulation	Sounds	Acq: 40%<br>Test: 42%
Dunsmoor et al. [53]	Humans (*N* = 36)	Differential cue	5	Electric stimulation	Faces	Acq: 60%<br>Test: 33%
Dowd et al. [54]	Humans (*N* = 28)	Differential cue	5	Electric stimulation	Geometrical shapes	Acq: 60%<br>Test: 4%
Gao et al. [55]	Humans (*N* = 53)	Differential cue	6	Electric stimulation	Geometrical shapes	Acq: 75%<br>Test: 75%
Glenn et al. [56]	Humans (*N* = 21)	Differential cue	4	Desperate female scream	Faces and bells	Acq.: 80%<br>Test: 0%
Glenn et al. [56]	S1: Youth (*N* = 16)<br>S2: Youth (*N* = 20)	Differential cue	4	Desperate female scream	Faces and bells	Acq.: 80%<br>Test: 80%
Glenn et al. [57]	Children (*N* = 40)	Differential cue	1	Desperate female scream	Faces	Acq: 75%<br>Test: 75%
Greenberg et al. [58]	S1: Humans (*N* = 32)<br>S2: Humans (*N* = 25)	Differential cue	6	Electric stimulation	Geometrical shapes	Acq: instructed<br>Test: 50%
Hunt et al. [59]	Humans (*N* = 89)	Differential cue	3	Electric stimulation	Geometrical shapes	Acq: 50%<br>Test: n.a.
Klein et al. [60]	Humans (*N* = 61)	Differential cue	5	Alarm sounds	Colored cartoons	Acq: 80%<br>Test: n.a.
Klein et al. [61]	Youth (*N* = 74)	Differential cue	9	Alarm sound	Colored cartoons	Acq: 100%<br>Test: 0%
Lange et al. [15]	Humans (*N* = 94)	Single cue	5	Electric stimulation	Geometrical shapes	Acq: 66%

						Test: 50%
Lee et al. [62]	Humans (*N* = 71)	Single vs. Differential cue	10	Electric stimulation	Geometrical shapes	Acq: 75%<br>Test: 75%
Li et al. [63]	Humans (*N* = 66)	Differential cue	8	Electric stimulation	Words	Acq: 50%<br>Test: 0%
Lissek et al. [58]	Humans (*N* = 38)	Differential cue	8	Electric stimulation	Geometrical shapes	Acq: 75%<br>Test: 50%
Lissek et al. [64]	Humans (*N* = 20)	Differential cue	8	Electric stimulation	Geometrical shapes	Acq: 75%<br>Test: 50%
Manbeck et al. [65]	Humans (*N* = 71)	Differential cue	3	Electric stimulation	Geometrical shapes	Acq: 50%<br>Test: 75%
Michalska et al. [66]	Children (*N* = 48)	Differential cue	9	Sound	Colored cartoons	Acq: 80%<br>Test: 0%
Niederstrasser et al. [67]	Humans (*N* = 48)	Differential cue	5	Electric stimulation	Movements of the harm	Acq: 75%<br>Test: 50%
Philips et al. [68]	Humans (*N* = 21)	Single cue	4	Sound	Colored words	Acq: 100%<br>Test: 0%
Reinhard et al. [69]	Youth (*N* = 79)	Differential cue	4	Desperate female scream	Faces	Acq: 83%<br>Test: 50%
Roesmann et al. [70]	Humans (*N* = 90)	Differential cue	7	Phobic pictures	Gabors	Acq: 33%<br>Test: 33%
Struyf et al. [71]	Humans (*N* = 84)	Differential cue	2	Electric stimulation	Faces	Acq: 75%<br>Test: 0%
Torrents-Rodas et al. [72]	Humans (*N* = 114)	Differential cue	4	Electric stimulation	Geometrical shapes	Acq: 50%<br>Test: 50%
Vandael et al. [73]	Humans (*N* = 50)	Differential cue	5	Electric stimulation	Colored lights	Acq: 100%<br>Test: n.a.

Vandael et al. [74]	Humans (*N* = 50)	Differential cue	5	Electric stimulation	Movement trajectory	Acq: 75%<br>Test: 50%
Van Meurs et al. [75]	Humans (*N* = 50)	Differential cue	6	Electric stimulation	Geometrical shapes	Acq: 50%<br>Test: 50%
Vervliet et al. [76]	S1: Humans (*N* = 58)<br>S2: Humans (*N* = 46)	Differential cue	6	Aversive pictures	Geometrical shapes	Acq: 100%<br>Test: 0%
Wong et al. [77]	Humans (*N* = 139)	Differential cue	7	Electric stimulation	Geometrical shapes	Acq: 75%<br>Test: n.a.
Wong et al. [78]	Humans (*N* = 113)	Differential cue	8	Electric stimulation	Geometrical shapes	Acq: 75%<br>Test: 0%
Wong et al. [79a]	Humans (*N* = 63)	Differential cue	7	Electric stimulation	Geometrical shapes	Acq: 75%<br>Test: 0%
Wong et al. [80b]	Humans (*N* = 64)	Differential cue	9	Electric stimulation	Animal pictures	Acq: 75%<br>Test: n.a.
Wong et al. [81]	Humans (*N* = 84)	Differential cue	7	Electric stimulation	Geometrical shapes	Acq: 75%<br>Test: n.a.
Zoladz et al. [82]	Humans (*N* = 291)	Differential cue	7	Air-blast	Geometrical shapes	Acq: 100%<br>Test: 0%
Studies examining the strength of generalization on the group level: Quadratic and (difference of) Gaussian contrast comparisons						
Antov et al. [83]	Humans (*N* = 19)	Differential cue	6	White noise	Gabor	Acq.: 100%<br>Test: 0%
Friedl et al. [84]	Humans (*N* = 51)	Differential cue	4	Noxious noise	Gabors	Acq: 100%<br>Test: n.a.
McTeague et al. [85]	Humans (*N* = 15)	Differential cue	6	White noise	Gabor	Acq.: 100%<br>Test: 0%
Plog et al. [86]	Humans (*N* = 40)	Differential cue	4	Sound	Gabors	Acq: 66%<br>Test: n.a.
Stegmann et al. [87]	Humans (*N* = 67)	Differential cue	4	Desperate female scream	Faces	Acq.: 80%<br>Test: 40%

Studies examining the strength of generalization on the group level: Hierarchical models including quadratic terms						
Ginat-Frohlich et al. [88]	Children (*N* = 46)	Differential cue	11	White noise	Colored cartoons	Acq.: 80%<br>Test: n.a.
Ginat-Frohlich et al. [89]	Children (*N* = 70)	Differential cue	9	White noise	Colored cartoons	Acq.: 80%<br>Test: 0.1%
Keefe et al. [90]	Humans (*N* = 108)	Differential cue	3	Electric stimulation	Geometrical shapes	Acq: 100%<br>Test: n.a.
Lommen et al. [91]	Humans (*N* = 78)	Differential cue	6	Electric stimulation	Geometrical shapes	Acq: 100%<br>Test: 100%
Meulders et al. [92]	Humans (*N* = 60)	Differential cue	5	Electric stimulation	Movement of the harm	Acq.: 75%<br>Test: 75%
Studies examining the strength of generalization on the group level: Various types of regression models						
Dos Santos et al. [93]	Rats (*N* = 232)	Single context	1	Electric stimulation	Cage	Acq.: 100%<br>Test: n..a.
Gao et al. [94]	Humans (*N* = 53)	Differential cue	6	Electric stimulation	Geometrical shapes	Acq: 75%<br>Test: 75%
Fan et al. [95]	Humans (*N* = 88)	Differential cue	4	Electric stimulation	Animal pictures	Acq: 100%<br>Test: 0%
Michalska et al. [66]	Children (*N* = 48)	Differential cue	9	White noise	Colored cartoons	Acq: 80%<br>Test: 0%
Nelson et al. [96]	Humans (*N* = 48)	Single cue	3	Electric stimulation	Geometrical shapes	Acq.: 100%<br>Test: 100%
Schroijen et al. [97]	Humans (*N* = 43)	Differential cue	4	Breathing occlusion	Breathing pressure	Acq: 88%<br>Test: 100%
Struyf et al. [8]	Humans (*N* = 168)	Differential cue	8	Aversive pictures	Geometrical shapes	Acq: 100%<br>Test: n.a.
Yu et al. [98]	Humans (*N* = 40)	Single and differential cue	10	Electric stimulation	Geometrical shapes	Acq: 50%<br>Test: n.a.
Zaman et al. [99]	Human (*N* = 60)	Single cue	3	Electric stimulation	Interoceptive sensation	Acq.: 75%<br>Test: n.a.
Studies reporting an index to quantify fear generalization, but without considering the generalization stimuli						

Kass et al. [100]	Mice (*N* = 66)	Single cue	4	Electric stimulation	Odors	Acq.: 100%<br>Test: 0%
Kopp et al. [101]	Humans (*N* = 48)	Differential cue	9	Electric stimulation	Phobic animals	Acq.: 80%<br>Test: 0%
Liu et al. [102]	Rabbits (*N* = 40)	Both differential and single cue	4	Electric stimulation	Sounds	Acq: 50%-100%<br>Test: n.a.
Miasknikov et al. [103]	Children (*N* = 59)	Differential cue	9	Sound	Colored cartoons	Acq: 80%<br>Test: n.a.
Scarlata et al. [104]	S1: Mice (*N* = 27)<br>S2: Mice (*N* = 12)	Single cue	1	Electric stimulation	Sounds	Acq: 100%<br>Test: 0%
Torrents-Rodas et al. [105]	Humans (*N* = 71)	Differential cue	8	Electric stimulation	Geometrical shapes	Acq: 75%<br>Test: 50%
You et al. [106]	Humans (*N* = 29)	Differential cue	30	Picture + audio of vomiting	Odors	Acq: 100%<br>Test: 71.4%
Studies reporting a discrimination index: e.g. just noticeable differences (JND)						
Dou et al. [107]	Humans (*N* = 63)	Differential cue	4	Electric stimulation	Faces	Acq.: 75%<br>Test: 50%
El-Bar et al. [43]	Youth (*N* = 40)	Differential cue	12	Loss of points	Sounds	Acq: 100%<br>Test: 0%
Holt et al. [108]	Humans (*N* = 47)	Differential cue	5	Electric stimulation	Faces	Acq.: 63%<br>Test: 100%
Levine et al. [109]	Humans (*N* = 21)	Differential cue	30	Electric stimulation	Animals, Tools, Fruits/Vegetables	Acq.: 50%<br>Test: n.a.
Lim et al. [110]	Humans (*N* = 20)	Differential cue	9	Electric stimulation	Faces	Acq.: 50%<br>Test: n.a.
Shalev et al. [111]	Humans (*N* = 315)	Differential cue	1	Aversive pictures	Gabor patches + sounds	Acq: 100%<br>Test: n.a.
Sterpenich et al. [112]	Humans (*N* = 32)	Single cue	15	Sound	Faces	Acq.: 50%<br>Test: n.a.
Tuominen et al. [31]	Humans (*N* = 37)	Differential cue	5	Electric stimulation	Faces	Acq.: 62.5%<br>Test: 100%

Simulation analysis

We used empirically informed cases to generate 55 gradients as true scores (5 model cases × 11 gradients per case). The amount of generalization was calculated using LDS and GI as algorithmic solutions, the curvature parameters σ_{Gauss} of a Gaussian model fit, λ_{exp}, from an exponential model fit and the relative importance of the linear over the quadratic component $\%_{linquad}$ from a quadratic polynomial model fit. All models were fitted to the data of individual cases using the Least-Squares approach in combination with the "Bound Optimization by Quadratic Approximation" (bobyqa) algorithm as implemented in the nloptr package (version 2.0.3) for R 4.1.2 [113].

We calculated 1) intercorrelations across these five indices to assess to what degree they reflect the same construct and 2) the range of values spanned across generalization gradients as a measure of differentiation between generalization profiles. For comparison, we normalized the true score generalization indices across cases to the range from 0 to 1 by subtracting the minimum value and dividing the result by the range of values. 3) To measure the sensitivity of generalization indices to measurement noise, we added noise from a normal distribution (M = 0, SD see below) to every of the 6 ratings within each gradient. We defined a middle amount of noise based on the empirical standard deviation of the arousal ratings (Stegmann et al., [18] of the same stimuli within the same subject across the generalization phase (SD = 1.59). By doubling and halving this value, we created a total of three different noise levels ranging from low to medium to high. For each of these noise levels we simulated 50 pairs of noise-corrupted gradients, for which we calculated the five generalization indices as described above. Robustness against noise was then estimated using correlations between pairs of generalization profiles drawn from the same true scores at different levels of noise. Presence of systematic bias was determined by calculating the mean and standard deviation of differences between true scores and estimates of generalization indices, separated by levels of noise.

Table S2. *Normalized Range Covered by Each Generalization Index per Model Case*

Case	LDS	GI	σ_{Gauss}	λ_{exp}	$\%_{linquad}$
full	1	0.76	1	1	1
bottom	0.50	0.67	1	0.99	1
middle	0.50	0.47	0.99	0.99	1
middle low diff	0.25	0.26	1	0.98	1
top	0.50	0.36	1	0.99	1

Note. LDS = linear deviation score, GI = generalization index, Gauss = Gaussian model fit, exp = exponential model fit, linquad = quadratic polynomial model fit, % = relative importance of the linear compared to the quadratic term.

Figure S2. *Unsystematic Variance between Pairs of Gradients drawn from the Same True Scores for Various Generalization Indices Separated by Model Case and Noise Level*

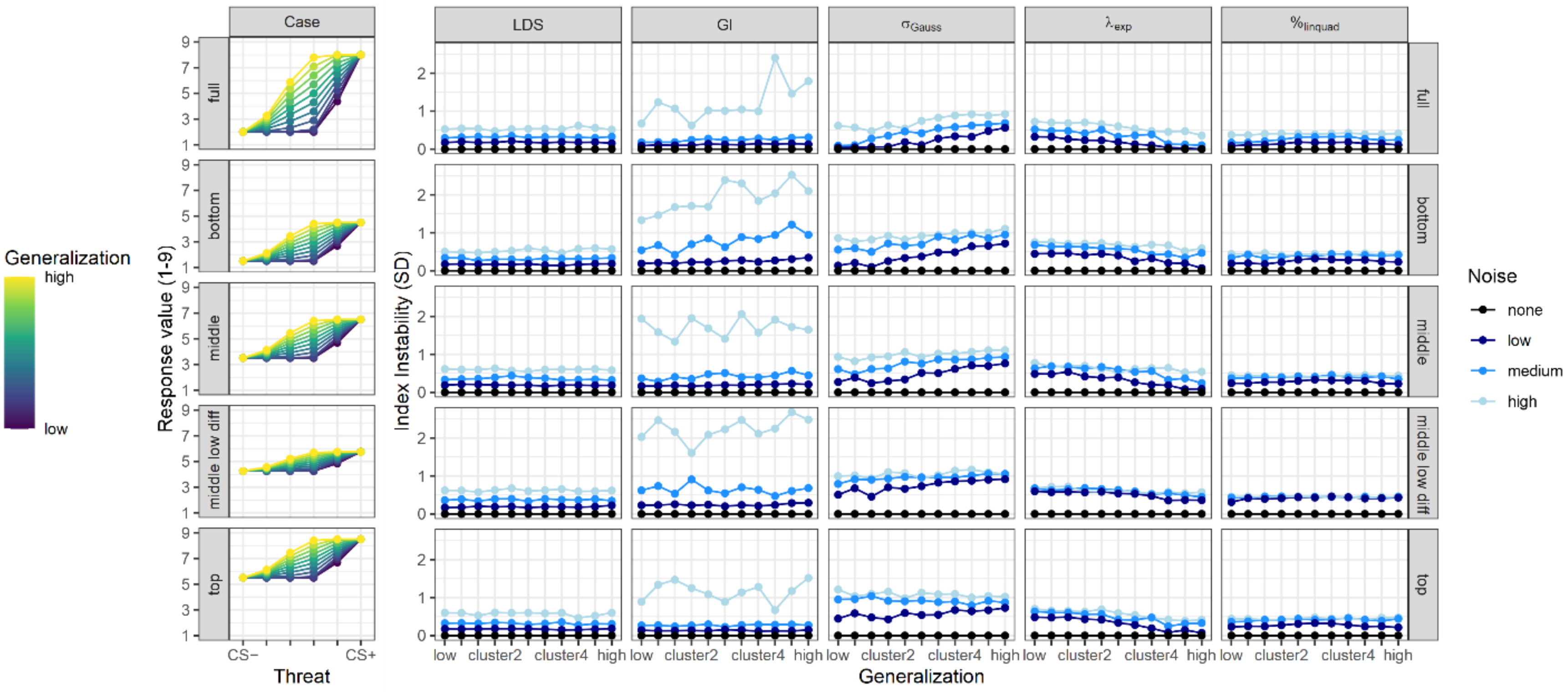

Note. LDS = linear deviation score, GI = generalization index, Gauss = Gaussian model fit, exp = exponential model fit, linquad = quadratic polynomial model fit, % = relative importance of the linear compared to the quadratic term. Please note, that the LDS and the λ_{exp} are inverted for better comparability.

Empirical analysis

Methods

Samples

The first sample consisted of 1,175 healthy participants, who completed a differential fear acquisition phase followed by a generalization test [18]. Exclusion criteria were left-handedness, non-Caucasian descent, intake of psychoactive medication, excessive consumption of alcohol, nicotine, and caffeine, consumption of illegal drugs, severe medical diseases, current and/or lifetime diagnosis of mental disorders, or being pregnant. The second sample (n = 256) was recruited using similar criteria and underwent the same experimental paradigm, except for a discrimination training between the first and second block of the generalization phase [20]. Due to the discrimination training, only the data of the first generalization block was considered for the analyses. Descriptive statistics of both samples are reported in Table S3.

Table S3. *Summary of the Sample Characteristics*

	Sample 1 (n = 1,175)				Sample 2 (n = 256)			
	M	*SD*	*Min*	*Max*	*M*	*SD*	*Min*	*Max*
Age (years)	25.7	5.9	18.0	50.0	24.0	5.2	18.0	50.0
STAI-T	34.6	8.2	20.0	67.0	35.7	8.6	20.0	59.0
ASI-3	12.1	8.3	0.0	48.0	15.1	9.5	0.0	48.0
ACQ	1.3	0.2	1.0	2.7	1.4	0.3	1.0	2.4
PSWQ	40.4	9.8	17.0	73.0	42.2	10.7	21.0	75.0
SPAI	33.2	17.0	0.0	103.7	36.1	18.0	2.7	118.0
LSAS	21.4	15.3	0.0	90.0	25.5	16.4	0.0	98.0
CTQ	32.1	8.1	25.0	97.0	32.9	10.1	25.0	87.0

Note. STAI-T = State-Trait Anxiety Inventory – Trait, ASI-3 = Anxiety Sensitivity Index 3, ACQ = Agoraphobic Cognition Questionnaire, PSWQ = Penn State Worry Questionnaire, SPAI = Social Phobia Anxiety Index, LSAS = Liebowitz Social Anxiety Scale, CTQ = Childhood Trauma Questionnaire.

All participants were screened for dimensional anxiety using the German version of the State-Trait Anxiety Inventory [114], the Anxiety Sensitivity Index 3 [115, 116], the Agoraphobic Cognitions Questionnaire [117, 118], the Liebowitz Social Anxiety Scale [119, 120], and the Social Phobia and Anxiety Inventory [121]. The Childhood Trauma [122, 123] was used for a retrospective assessment of childhood maltreatment. We calculated sum/mean scores for all questionnaires (for an illustration of the value distribution, see Figure S3). All volunteers gave written informed consent and were paid 50 Euros. All

procedures complied with the Declaration of Helsinki (Version 2008) and the studies were approved by the ethics committees of the involved Universities.

Figure S3. *Distribution of Questionnaire Scores Assessing Individual Differences in Anxiety and Related Constructs in the Full Sample (n = 1431)*

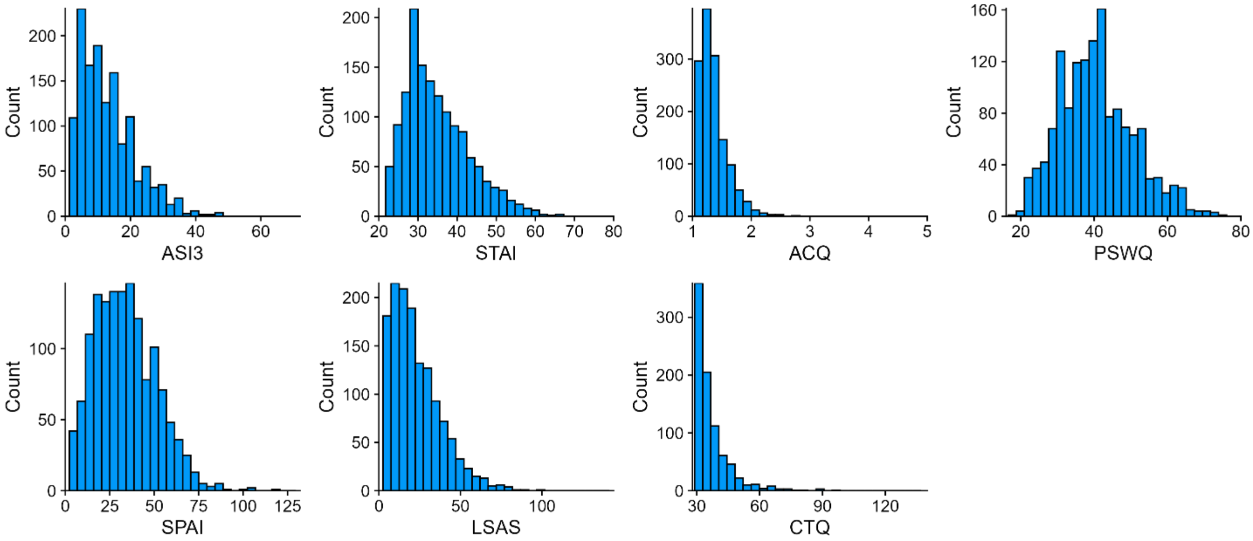

Note. Each plot displays the complete possible score range of the respective questionnaire. ASI-3 = Anxiety Sensitivity Index 3, STAI-T = State-Trait Anxiety Inventory – Trait, ACQ = Agoraphobic Cognition Questionnaire, PSWQ = Penn State Worry Questionnaire, SPAI = Social Phobia Anxiety Index, LSAS = Liebowitz Social Anxiety Scale, CTQ = Childhood Trauma Questionnaire.

Experimental protocol

Participants completed a differential fear acquisition and generalization protocol adapted from Lau, Lissek [124], in which two female faces with neutral expression served as CSs. The paradigm consisted of three phases: During *pre-acquisition*, each of the two faces was singularly presented four times for a duration of 6 s (in total eight trials). In the *acquisition phase*, both faces were presented 12 times each (24 trials in total). Ten presentations of one face (CS+) were followed by the US, consisting of a fearful facial expression of the same person with a simultaneous presentation of a 95 dB loud female scream for a duration of 1.5 s. Participants were not instructed about the CS-US contingencies and the assignment of faces to CS+ and CS− was counterbalanced across participants. During the generalization test, four generalization stimuli (GS) were presented in addition to the CSs. Generalization stimuli were morphs of the original CS faces in 20% steps. Each stimulus was presented 12 times (in total 72 trials). Half of CS+ presentations were paired with the US to prevent extinction of the conditioned responses. After the first half and at the end of the acquisition as well as of the generalization phase, participants were asked to

rate the faces regarding valence and arousal (both 9-point Likert-scales; from 1 = very unpleasant/ very calm to 9 = very pleasant/ very arousing) as well as US-contingency (11-point Likert-scale; from 0 to 100% in 10% increments). Please note that valence ratings were inverted for the current analyses to increase comparability. Skin conductance was continuously recorded throughout the whole experimental paradigm.

Physiological data processing

Skin conductance was measured at the thenar and hypothenar eminences of the participant's non-dominant hand with Ag/AgCl electrodes, using a constant-voltage system (0.5 V). Signals were amplified and recorded using a V-Amp-16 and Vision Recorder software (Brainproducts, Gilching, Germany) at a sampling rate of 1,000 Hz. Offline data processing within the Vision Analyzer 2 software (Brainproducts, Gilching, Germany) included filtering with a high cutoff filter of 1 Hz and a notch filter of 50 Hz. Skin conductance responses to CS+, CS- and the GS were analyzed by quantifying SCR amplitudes as the base-to-peak difference in µS between response onset (900–4,000 ms after stimulus onset) and the first peak after response onset [2,000–6,000 ms after stimulus onset; 125]. A minimum response criterion of 0.02 µS was applied, with lower responses scored as 0. To compensate for the skewed distribution of SCR amplitudes, we employed a square root transformation.

Results

Table S4. *Summary of Generalization Indices and Model Parameters of Study 1 (n = 1,175) and Study 2 (n = 256)*

	Study 1																			
	Arousal ratings					Skin conductance responses					Unpleasantness ratings					US expectancy ratings				
	M	*SD*	*Min*	*Max*	NAs	*M*	*SD*	*Min*	*Max*	NAs	*M*	*SD*	*Min*	*Max*	NAs	*M*	*SD*	*Min*	*Max*	NAs
Level	3.78	1.27	1.00	7.83	0	0.10	0.09	0.00	0.67	0	4.49	1.05	1.17	8.08	0	3.94	1.51	1.00	9.58	0
Diff	3.27	2.30	-5.00	8.00	0	0.05	0.11	-0.30	1.05	0	2.76	2.41	-5.00	8.00	0	6.00	2.60	-5.00	10.00	0
LDS	0.70	1.09	-4.25	5.62	0	0.01	0.05	-0.17	0.32	0	0.75	1.07	-3.12	5.00	0	1.77	1.38	-2.75	7.25	0
GI	2.57	1.15	0.56	15.00	0	4.60	5.55	0.00	92.00	126	2.81	1.03	0.44	11.20	0	1.71	0.87	0.36	7.00	0
Gaussian model fit:																				
σ	2.22	2.81	0.20	14.30	0	4.71	2.58	0.20	14.40	0	2.18	2.85	0.20	13.60	0	1.32	1.61	0.19	13.20	0
b	54.80	170.00	-1000	1000	0	-0.21	3.81	-43.5	20.30	0	45.1	153.00	-1000	1000	0	30.10	106.00	-1000	1000	0
a	0.69	6.41	-35.20	32.40	0	0.09	0.19	-0.71	1.77	0	1.87	6.07	-35.00	32.90	0	1.41	4.36	-40.30	37.50	0
R^2	0.83	0.18	0.00	1.00	18	0.27	0.27	0.00	1.00	42	0.75	0.29	0.00	1.00	30	0.92	0.14	0.00	1.00	13
Exponential model fit:																				
λ	0.56	0.57	0.00	2.00	0	0.11	0.23	0.00	1.14	0	0.58	0.59	0.00	1.94	0	0.82	0.62	0	2	0
b	6.51	11.80	0.00	62.40	0	1.45	1.41	0.00	3.36	0	5.42	10.30	0.00	55.60	0	3.80	9.62	0	67.50	0
a	-4.36	12.20	-59.20	6.85	0	-1.37	1.43	-3.21	0.41	0	-2.31	10.80	-55.10	7.34	0	-2.24	9.84	-65.80	9.58	0
R^2	0.80	0.22	0.00	1.00	101	0.35	0.30	0.00	0.99	357	0.77	0.24	0.00	1.00	144	0.90	0.14	0.01	1.00	25
Quadratic polynomial model fit:																				
c	0.15	0.22	-0.76	1.10	0	0.00	0.01	-0.03	0.06	0	0.16	0.21	-0.62	0.99	0	0.36	0.26	-0.54	1.43	0
b	-0.40	1.56	-7.72	5.87	0	-0.01	0.06	-0.38	0.24	0	-0.56	1.55	-6.97	5.26	0	-1.41	1.81	-10.00	5.64	0
a	2.90	2.35	-3.95	14.80	0	0.09	0.12	-0.20	0.79	0	4.06	2.51	-3.45	14.40	0	3.34	2.44	-3.60	18.60	0
$\%_{linquad}$	0.47	0.06	0.40	0.60	18	0.51	0.07	0.40	0.60	20	0.47	0.06	0.40	0.60	29	0.45	0.05	0.40	0.60	13
R^2	0.83	0.18	0.01	1.00	18	0.45	0.28	0.00	1.00	20	0.81	0.20	0.01	1.00	29	0.88	0.11	0.14	1.00	13

	**Study 2**																				
	Arousal ratings					Skin conductance responses					Unpleasantness ratings					US expectancy ratings					
	M	*SD*	*Min*	*Max*	NAs	*M*	*SD*	*Min*	*Max*	NAs	*M*	*SD*	*Min*	*Max*	NAs	*M*	*SD*	*Min*	*Max*	NAs	
Level	4.33	1.49	1.00	8.25	0	0.19	0.18	0.00	0.97	0	4.49	1.05	1.17	8.08	0	3.48	1.48	1.00	8.5	0	
Diff	3.93	2.12	-1.50	8.00	0	0.07	0.33	-1.45	1.14	0	3.30	2.52	-7.50	8.00	0	5.80	2.61	-4.50	10.00	0	
LDS	0.62	1.11	-3.62	4.12	0	0.01	0.18	-0.49	0.72	0	0.37	1.12	-3.00	3.88	0	1.59	1.57	-5	5	0	
GI	2.51	0.90	0.56	5.17	0	4.94	6.49	0.00	51.6	85	3.10	2.21	0.50	28.00	0	1.80	1.56	0.36	12.60	0	
	Gaussian model fit:																				
σ	2.38	2.58	0.20	10.80	0	4.43	3.21	0.20	12.30	0	3.22	3.37	0.20	12.00	0	1.19	1.29	0.20	10.40	0	
b	71.20	160.00	-90.50	1000	0	-5.01	46.00	-390	105	0	98.60	207.0	-372.0	1000	0	29.70	96.20	-7.02	1000	0	
a	0.25	6.57	-37.30	8.76	0	0.32	1.57	-3.39	13.40	0	-0.16	8.20	-38.30	14.90	0	1.03	3.60	-32.80	7.87	0	
R^2	0.88	0.18	0.06	1.00	7	0.26	0.27	0.00	1.00	16	0.78	0.27	0.00	1.00	5	0.92	0.15	0.00	1.00	1	
	Exponential model fit:																				
λ	0.51	0.57	0.00	1.86	0	0.13	0.29	0.00	1.23	0	0.45	0.56	0.00	1.87	0	0.82	0.65	0.00	1.91	0	
b	9.23	13.60	0.00	63.30	0	1.76	1.53	0.00	10.30	0	9.52	13.60	0.00	68.20	0	4.56	10.50	0.00	63.20	0	
a	-6.91	14.00	-63.00	7.53	0	-1.63	1.56	-10.40	0.64	0	-6.44	14.20	-66.60	6.99	0	-3.46	10.60	-62.40	6.29	0	
R^2	0.85	0.19	0.09	1.00	10	0.28	0.27	0.00	0.95	96	0.78	0.25	0.00	1.00	21	0.88	0.17	0.05	1.00	4	
	Quadratic polynomial model fit:																				
c	0.13	0.22	-0.74	0.78	0	0.00	0.03	-0.14	0.13	0	0.07	0.22	-0.61	0.75	0	0.34	0.29	-1.03	0.89	0	
b	-0.12	1.59	-5.54	6.29	0	0.01	0.25	-1.10	0.94	0	0.14	1.57	-5.72	5.64	0	-1.26	1.92	-5.59	7.56	0	
a	2.80	2.43	-4.85	13.80	0	0.15	0.42	-0.84	2.17	0	3.23	2.45	-4.10	14.40	0	2.71	2.19	-5.55	11.20	0	
$\%_{linquad}$	0.48	0.06	0.40	0.60	6	0.50	0.07	0.40	0.60	16	0.49	0.06	0.40	0.60	5	0.45	0.05	0.40	0.60	1	
R^2	0.86	0.17	0.03	1.00	6	0.38	0.25	0.00	0.97	16	0.82	0.19	0.09	1.00	5	0.85	0.13	0.12	1.00	1	

Note. NAs = number of missing values, LDS = linear deviation score, GI = generalization index, R^2 = explained variance of the individual generalization gradients. Note that missing values in R^2 are due to either the observed or the estimated data being on a flat line resulting in zero variance, linquad = quadratic polynomial model fit, % = relative importance of the linear compared to the quadratic term.

Figure S4. *Correlations Between Different Indices of Fear Generalization (Panel A) and their Correlations with Basic Indices of Threat Responsiveness (Panel B) for Unpleasantness and US Expectancy Ratings.*

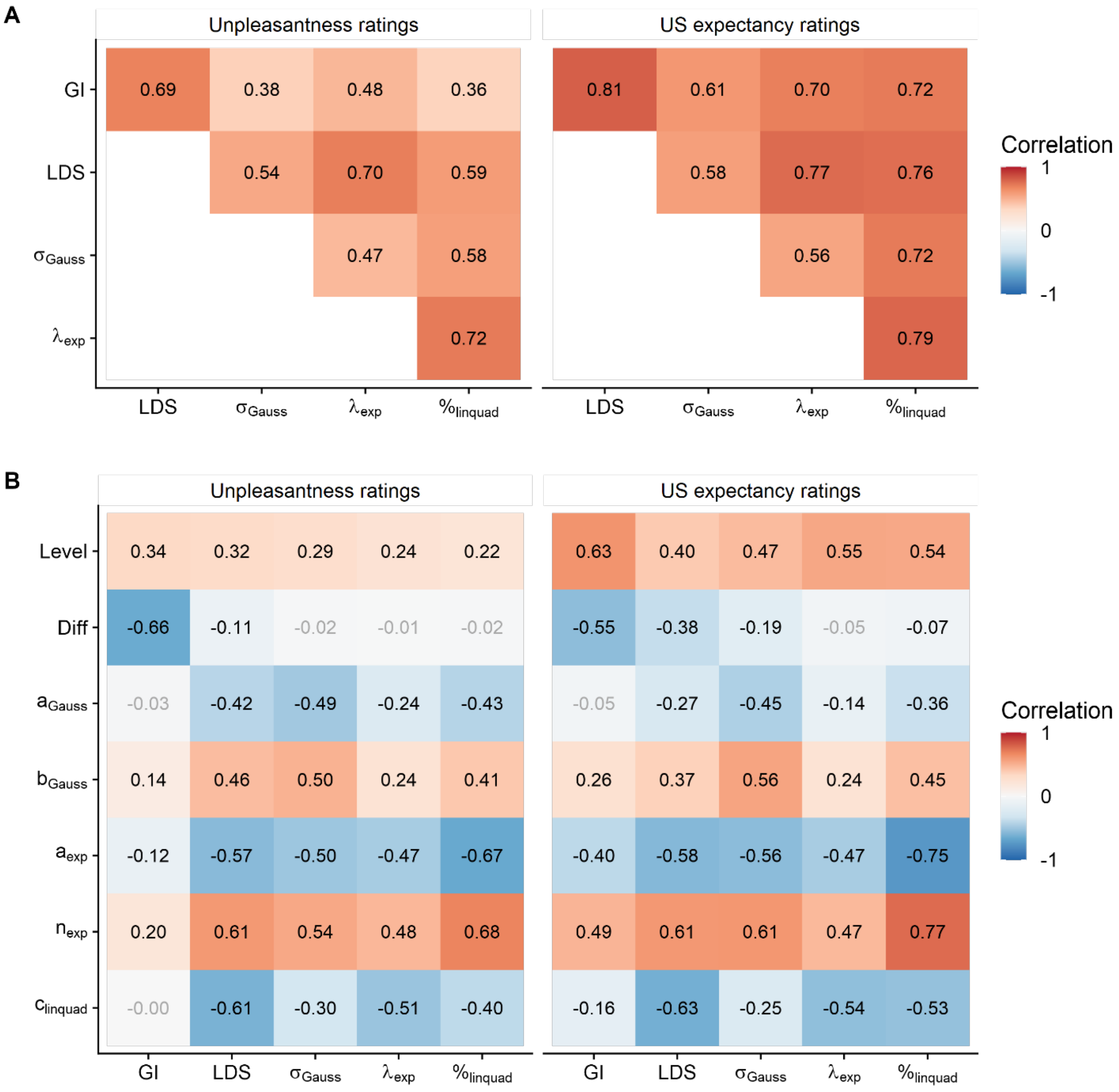

Note. GI = generalization index, LDS = linear deviation score, Gauss = Gaussian model fit, exp = exponential model fit, linquad = quadratic polynomial model fit, % = relative importance of the linear compared to the quadratic term, Level = mean response level, Diff = CS differentiation. Statistically significant correlations are printed in black. Please note that the LDS and the λ_{exp} are inverted for better comparability.

Figure S5. *Correlations Between Different Measures of the First and Second Half of the Fear Generalization Phase for Unpleasantness and US Expectancy Ratings*

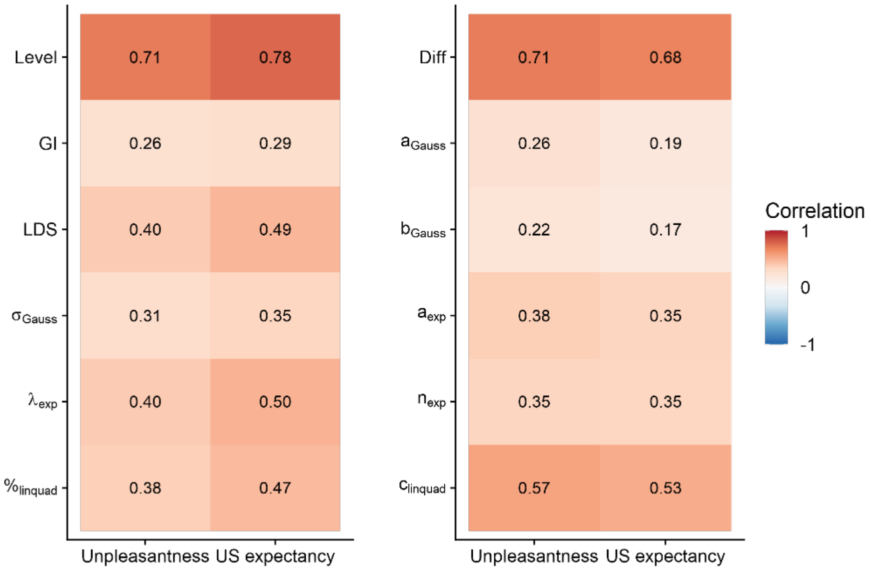

Note. GI = generalization index, LDS = linear deviation score, Gauss = Gaussian model fit, exp = exponential model fit, linquad = quadratic polynomial model fit, % = relative importance of the linear compared to the quadratic term, Level = mean response level, Diff = CS difference. Statistically significant correlations are printed in black. Please note that the LDS and the λ_{exp} are inverted for better comparability.

Figure S6. *Correlations Between Anxiety-Related Constructs and Indices of Fear Generalization (Panel A) as well as Basic Indices of Fear Responsiveness (Panel B) for Unpleasantness and US Expectancy Ratings*

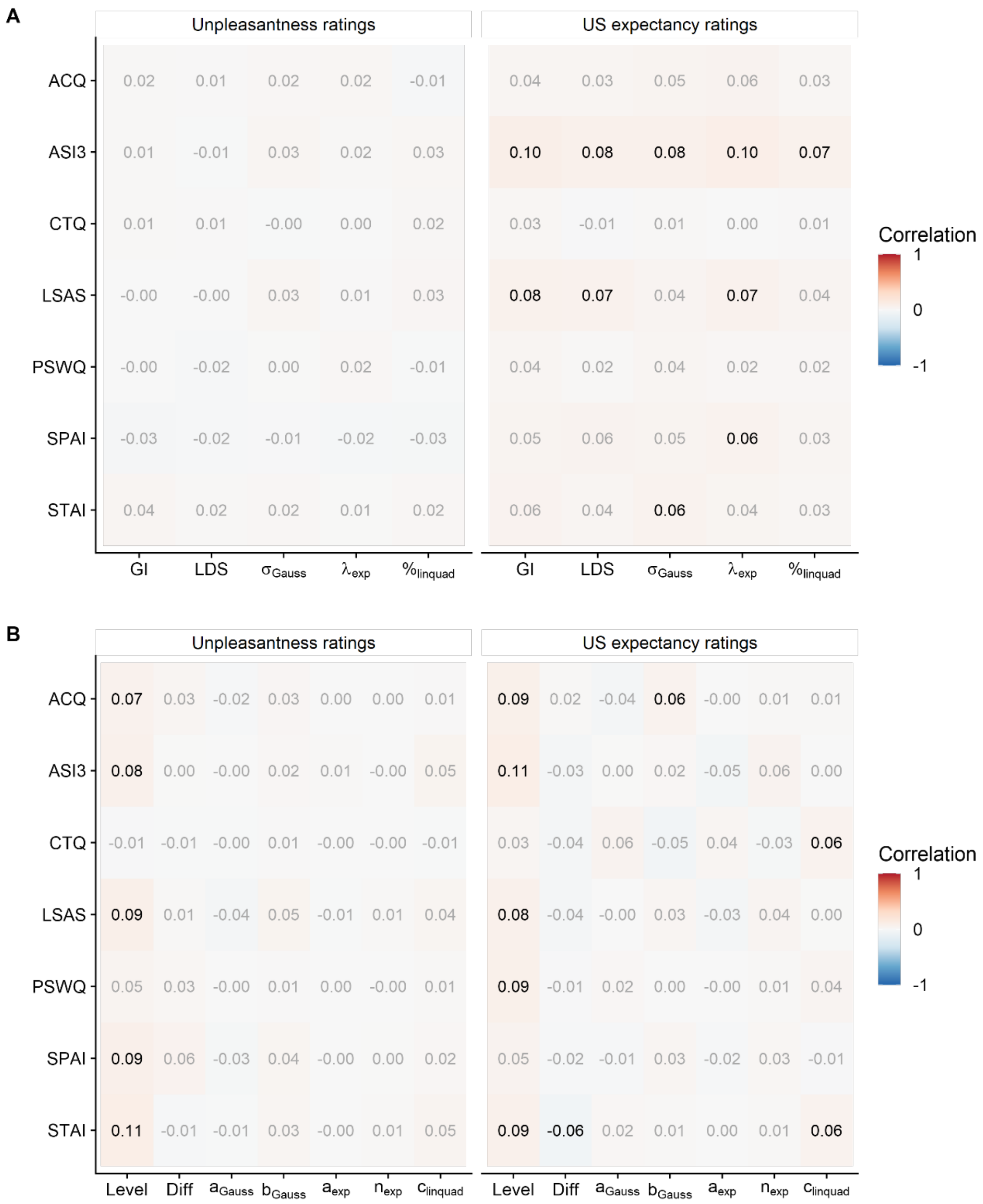

Note. ACQ = Agoraphobic Cognition Questionnaire, ASI-3 = Anxiety Sensitivity Index 3, CTQ = Childhood Trauma Questionnaire, LSAS = Liebowitz Social Anxiety Scale, PSWQ = Penn State Worry Questionnaire, SPAI = Social Phobia Anxiety Index, STAI-T = State-Trait Anxiety Inventory – Trait, GI = generalization index, LDS = linear deviation score, Gauss = Gaussian model fit, exp = exponential model fit, linquad = quadratic polynomial model fit, % = relative importance of the linear compared to the quadratic term, Level = mean response level, Diff = CS differentiation. Statistically significant correlations are printed in black. Please note that the LDS and the λ_{exp} are inverted for better comparability.

Figure S7. *Comparison of Correlations Among Gradient Curvature Parameters Between the First Sample and the Second Sample*

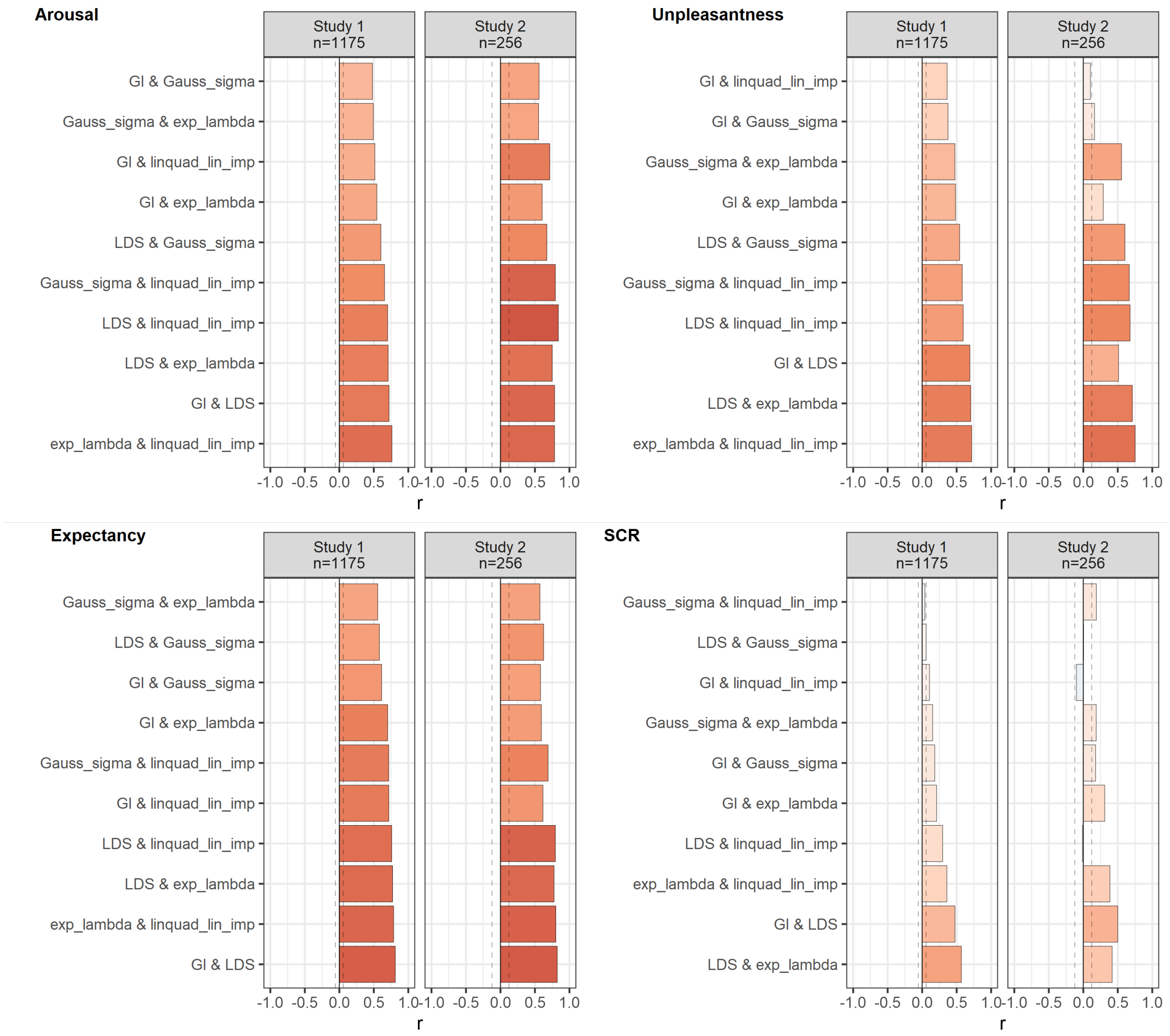

Note. GI = generalization index, LDS = linear deviation score, Gauss = Gaussian model fit, exp = exponential model fit, linquad = quadratic polynomial model fit, lin_imp = relative importance of the quadratic compared to the linear term. Dashed lines indicate statistical significance at $p < .05$.

Figure S8. *Comparison of Correlations Among Gradient Curvature Parameters and Basic Parameters of Fear Generalization Between the First Sample and the Second Sample*

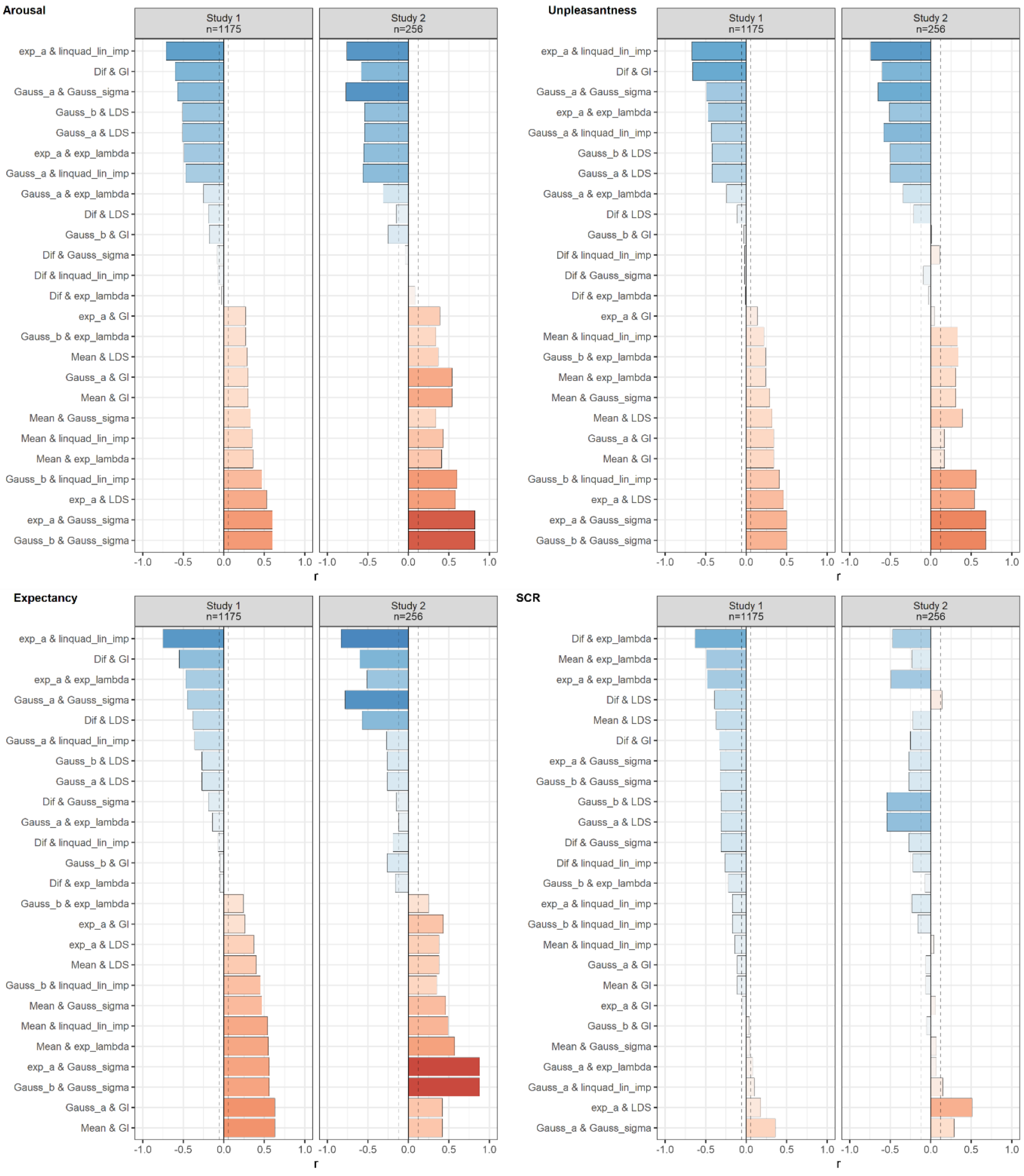

Note. GI = generalization index, LDS = linear deviation score, Gauss = Gaussian model fit, exp = exponential model fit, linquad = quadratic polynomial model fit, lin_imp = relative importance of the quadratic compared to the linear term, Mean = mean response level, Dif = CS difference. Dashed lines indicate statistical significance at $p < .05$.

Figure S9. *Comparison of Correlations Among Gradient Curvature Parameters and Individual Differences in Anxiety-Related Constructs Between the First Sample and the Second Sample.*

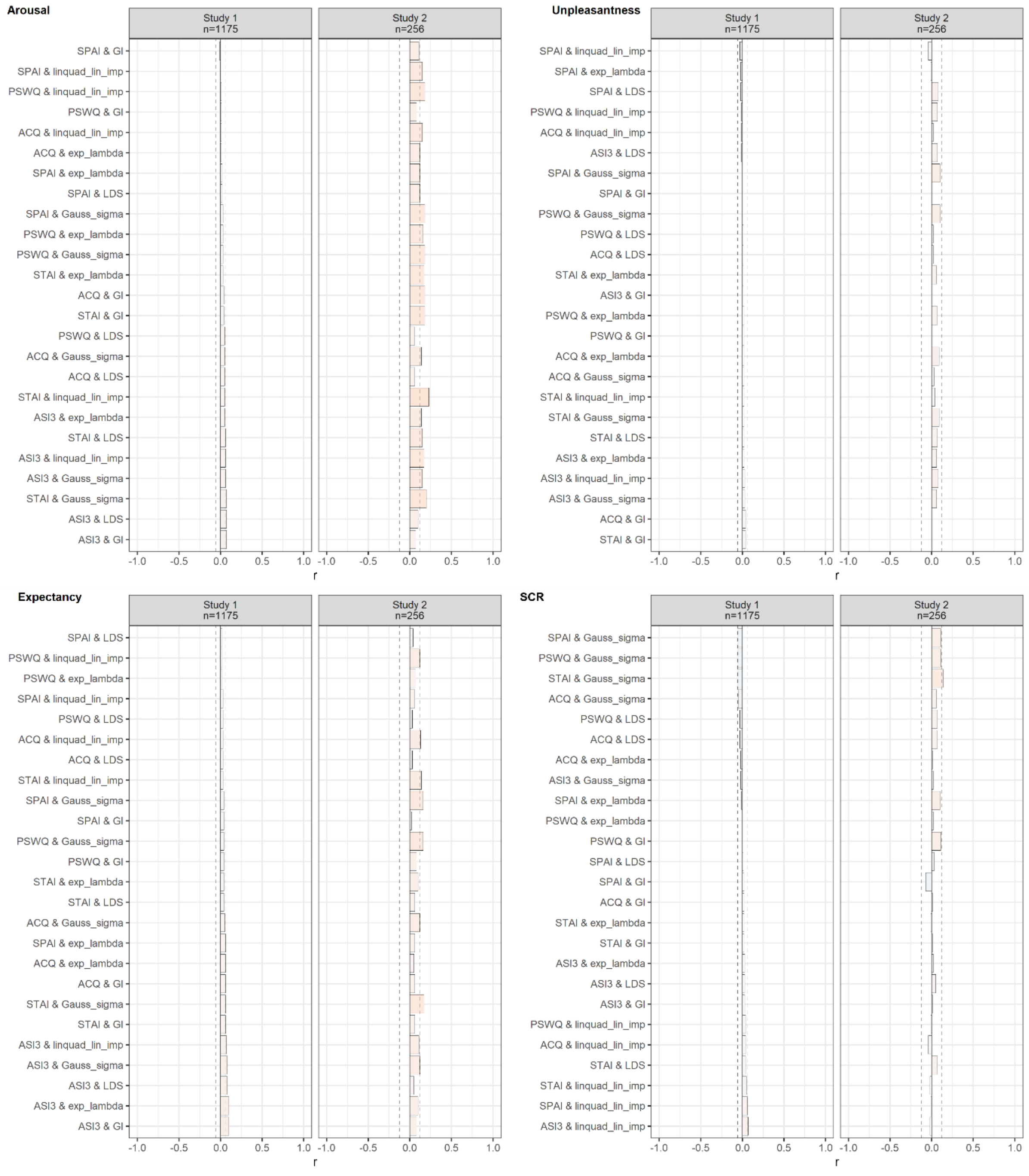

Note. GI = generalization index, LDS = linear deviation score, Gauss = Gaussian model fit, exp = exponential model fit, linquad = quadratic polynomial model fit, lin_imp = relative importance of the quadratic compared to the linear term, ACQ = Agoraphobic Cognition Questionnaire, ASI-3 = Anxiety Sensitivity Index 3, PSWQ = Penn State Worry Questionnaire, SPAI = Social Phobia Anxiety Index, STAI-T = State-Trait Anxiety Inventory – Trait. Dashed lines indicate statistical significance at $p < .05$.

Figure S10. *Comparison of Correlations Among Basic Parameters and Individual Differences in Anxiety-Related Constructs Between the First Sample and the Second Sample*

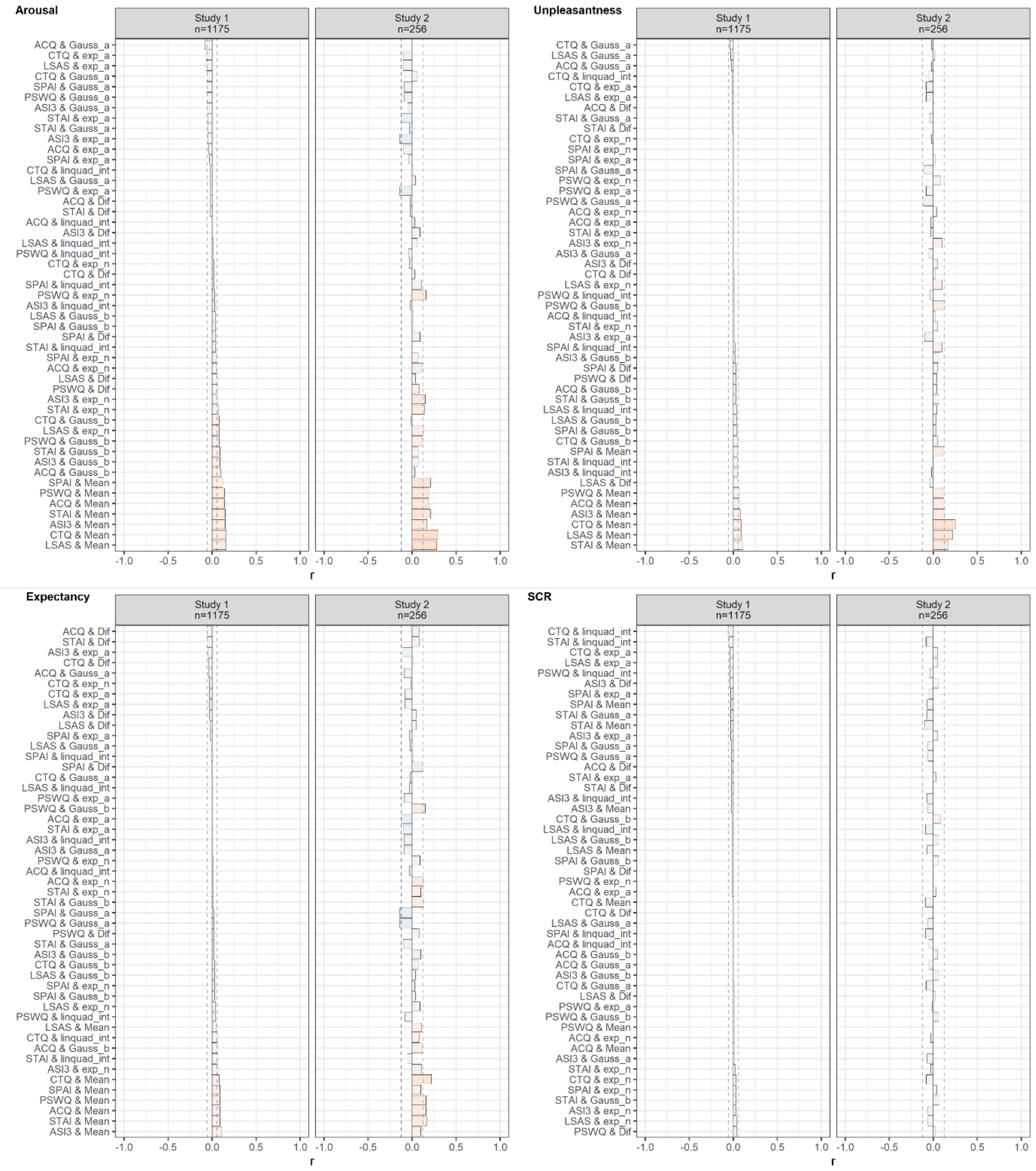

Note. Mean = mean response level, Dif = CS difference, Gauss = Gaussian model fit, exp = exponential model fit, linquad = quadratic polynomial model fit, ACQ = Agoraphobic Cognition Questionnaire, ASI-3 = Anxiety Sensitivity Index 3, CTQ = Childhood Trauma Questionnaire, LSAS = Liebowitz Social Anxiety Scale, PSWQ = Penn State Worry Questionnaire, SPAI = Social Phobia Anxiety Index, STAI-T = State-Trait Anxiety Inventory – Trait. Dashed lines indicate statistical significance at $p < .05$.

Supplemental References

1. Moher, D., et al., *Preferred reporting items for systematic review and meta-analysis protocols (PRISMA-P) 2015 statement.* Systematic Reviews, 2015. **4**(1): p. 1.
2. Radua, J., *PRISMA 2020 – An updated checklist for systematic reviews and meta-analyses.* Neuroscience & Biobehavioral Reviews, 2021. **124**: p. 324-325.
3. Lissek, S., et al., *Generalized anxiety disorder is associated with overgeneralization of classically conditioned fear.* Biol Psychiatry, 2014. **75**(11): p. 909-15.
4. Lenaert, B., et al., *Generalization on the Basis of Prior Experience Is Predicted by Individual Differences in Working Memory.* Behavior Therapy, 2016. **47**(1): p. 130-140.
5. Kausche, F.M., et al., *Neural signature of delayed fear generalization under stress.* Psychophysiology, 2021. **58**(11): p. e13917.
6. Onat, S. and C. Buchel, *The neuronal basis of fear generalization in humans.* Nat Neurosci, 2015. **18**(12): p. 1811-8.
7. Resnik, J. and R. Paz, *Fear generalization in the primate amygdala.* Nature Neuroscience, 2015. **18**(2): p. 188-190.
8. Struyf, D., et al., *Gradients of fear: How perception influences fear generalization.* Behaviour Research and Therapy, 2017. **93**: p. 116-122.
9. Cha, J., et al., *Circuit-Wide Structural and Functional Measures Predict Ventromedial Prefrontal Cortex Fear Generalization: Implications for Generalized Anxiety Disorder.* The Journal of Neuroscience, 2014. **34**(11): p. 4043.
10. Dunning, J.P. and G. Hajcak, *Gradients of Fear Potentiated Startle During Generalization, Extinction, and Extinction Recall--and Their Relations With Worry.* Behav Ther, 2015. **46**(5): p. 640-51.
11. Feldman, B.E., *Relative importance and value.* Available at SSRN 2255827, 2005.
12. Aslanidou, A., et al., *No influence of threat uncertainty on fear generalization.* Psychophysiology, 2024. **61**(1): p. e14423.
13. Imholze, C., et al., *Prediction of Changes in Negative Affect During the COVID-19 Pandemic by Experimental Fear Conditioning and Generalization Measures.* Zeitschrift für Psychologie, 2023. **231**(2): p. 137-148.
14. Kaczkurkin, A.N., et al., *Neural Substrates of Overgeneralized Conditioned Fear in PTSD.* Am J Psychiatry, 2017. **174**(2): p. 125-134.
15. Lange, I., et al., *Functional neuroimaging of associative learning and generalization in specific phobia.* Progress in Neuro-Psychopharmacology and Biological Psychiatry, 2019. **89**: p. 275-285.

16. Lange, I., et al., *Behavioral pattern separation and its link to the neural mechanisms of fear generalization.* Social Cognitive and Affective Neuroscience, 2017. **12**(11): p. 1720-1729.
17. Reutter, M. and M. Gamer, *Individual patterns of visual exploration predict the extent of fear generalization in humans.* Emotion, 2023. **23**(5): p. 1267-1280.
18. Stegmann, Y., et al., *Individual differences in human fear generalization—pattern identification and implications for anxiety disorders.* Translational Psychiatry, 2019. **9**(1): p. 307.
19. Zhu, X., et al., *Sequential fear generalization and network connectivity in trauma exposed humans with and without psychopathology.* Communications Biology, 2022. **5**(1): p. 1275.
20. Herzog, K., et al., *Reducing Generalization of Conditioned Fear: Beneficial Impact of Fear Relevance and Feedback in Discrimination Training.* Frontiers in Psychology, 2021. **12**.
21. Mertens, G., V. Bouwman, and I.M. Engelhard, *Conceptual fear generalization gradients and their relationship with anxious traits: Results from a Registered Report.* International Journal of Psychophysiology, 2021. **170**: p. 43-50.
22. Reinhard, J., et al., *Fear conditioning and stimulus generalization in association with age in children and adolescents.* European Child & Adolescent Psychiatry, 2022. **31**(10): p. 1581-1590.
23. Spruyt, A., et al., *Feature-Specific Attention Allocation Modulates the Generalization of Recently Acquired Likes and Dislikes.* Experimental Psychology, 2014. **61**(2): p. 85–98.
24. Dou, H., et al., *Impact of observational and direct learning on fear conditioning and generalization in humans.* Progress in Neuro-Psychopharmacology and Biological Psychiatry, 2023. **121**: p. 110650.
25. Grosso, A., et al., *A neuronal basis for fear discrimination in the lateral amygdala.* Nature Communications, 2018. **9**(1): p. 1214.
26. Huang, X., et al., *The overgeneralization of pain-related fear in individuals with higher pain sensitivity: A behavioral and event-related potential study.* Brain Research, 2023. **1818**: p. 148473.
27. Kampermann, L., et al., *Fixation-pattern similarity analysis reveals adaptive changes in face-viewing strategies following aversive learning.* eLife, 2019. **8**: p. e44111.
28. Kausche, F.M., et al., *Noradrenergic stimulation increases fear memory expression.* European Neuropsychopharmacology, 2021. **43**: p. 71-81.
29. Kausche, F.M., et al., *Acute stress leaves fear generalization in healthy individuals intact.* Cognitive, Affective, & Behavioral Neuroscience, 2021. **21**(2): p. 372-389.
30. Porter, D.B., et al., *Aversive outcomes impact human olfactory discrimination learning and generalization.* Behavioral Neuroscience, 2021. **135**(5): p. 642-653.

31. Tuominen, L., et al., *Neural Abnormalities in Fear Generalization in Schizophrenia and Associations With Negative Symptoms.* Biological Psychiatry: Cognitive Neuroscience and Neuroimaging, 2021. **6**(12): p. 1165-1175.
32. Tuominen, L., et al., *The relationship of perceptual discrimination to neural mechanisms of fear generalization.* NeuroImage, 2019. **188**: p. 445-455.
33. Wang, J., et al., *Common and separable behavioral and neural mechanisms underlie the generalization of fear and disgust.* Progress in Neuro-Psychopharmacology and Biological Psychiatry, 2022. **116**: p. 110519.
34. Yu, K., et al., *The assessment of gender differences in perceptual fear generalization and related processes.* Behaviour Research and Therapy, 2024. **183**: p. 104640.
35. Zaman, J., K. Yu, and J.C. Lee, *Individual differences in stimulus identification, rule induction, and generalization of learning.* Journal of Experimental Psychology: Learning, Memory, and Cognition, 2023. **49**(6): p. 1004-1017.
36. Zaman, J., et al., *Perceptual errors are related to shifts in generalization of conditioned responding.* Psychol Res, 2021. **85**(4): p. 1801-1813.
37. Zaman, J., et al., *Direct and indirect effects of perception on generalization gradients.* Behaviour Research and Therapy, 2019. **114**: p. 44-50.
38. Zaman, J., et al., *Probing the role of perception in fear generalization.* Scientific Reports, 2019. **9**(1): p. 10026.
39. Zenses, A.-K., et al., *Differences in perceptual memory determine generalization patterns.* Behaviour Research and Therapy, 2021. **136**: p. 103777.
40. Cha, J., et al., *Clinically Anxious Individuals Show Disrupted Feedback between Inferior Frontal Gyrus and Prefrontal-Limbic Control Circuit.* The Journal of Neuroscience, 2016. **36**(17): p. 4708.
41. Cha, J., et al., *Hyper-Reactive Human Ventral Tegmental Area and Aberrant Mesocorticolimbic Connectivity in Overgeneralization of Fear in Generalized Anxiety Disorder.* The Journal of Neuroscience, 2014. **34**(17): p. 5855.
42. Dymond, S., et al., *Far from the threatening crowd: Generalisation of conditioned threat expectancy and fear in COVID-19 lockdown.* Learn Behav, 2024. **52**(3): p. 262-271.
43. El-Bar, N., et al., *Over-generalization in youth with anxiety disorders.* Social Neuroscience, 2017. **12**(1): p. 76-85.
44. Hammell, A.E., et al., *The temporal course of over-generalized conditioned threat expectancies in posttraumatic stress disorder.* Behaviour Research and Therapy, 2020. **124**: p. 103513.

45. Laufer, O., D. Israeli, and R. Paz, *Behavioral and Neural Mechanisms of Overgeneralization in Anxiety.* Current Biology, 2016. **26**(6): p. 713-722.
46. Wickens, D.D., H.M. Schroder, and J.D. Snide, *Primary stimulus generalization of the GSR under two conditions.* Journal of Experimental Psychology, 1954. **47**(1): p. 52-56.
47. Zaman, J., K. Yu, and S. Verheyen, *The idiosyncratic nature of how individuals perceive, represent, and remember their surroundings and its impact on learning-based generalization.* Journal of Experimental Psychology: General, 2023: p. No Pagination Specified-No Pagination Specified.
48. Zaman, J., et al., *Perceptual errors are related to shifts in generalization of conditioned responding.* Psychological Research, 2021. **85**(4): p. 1801-1813.
49. Ahmed, O. and P.F. Lovibond, *The Impact of Instructions on Generalization of Conditioned Fear in Humans.* Behavior Therapy, 2015. **46**(5): p. 597-603.
50. Cooper, S.E., et al., *Test–retest reliability of human threat conditioning and generalization across a 1-to-2-week interval.* Psychophysiology, 2023. **60**(6): p. e14242.
51. Davidson, P., et al., *A more generalized fear response after a daytime nap.* Neurobiology of Learning and Memory, 2018. **151**: p. 18-27.
52. Dunsmoor, J.E., et al., *Threat intensity widens fear generalization gradients.* Behavioral Neuroscience, 2017. **131**(2): p. 168-175.
53. Dunsmoor, J.E., S.R. Mitroff, and K.S. LaBar, *Generalization of conditioned fear along a dimension of increasing fear intensity.* Learning & Memory, 2009. **16**(7): p. 460-469.
54. Dowd, E.W., S.R. Mitroff, and K.S. LaBar, *Fear generalization gradients in visuospatial attention.* Emotion, 2016. **16**(7): p. 1011-8.
55. Gao, Y., et al., *Role of Cue Training, Context, and Stimulus Intensity on Fear Generalization in Humans.* Behav Sci (Basel), 2023. **13**(6).
56. Glenn, D.E., et al., *Social relevance modulates multivariate neural representations of threat generalization in children and adults.* Developmental Psychobiology, 2021. **63**(7): p. e22185.
57. Glenn, C.R., et al., *The development of fear learning and generalization in 8–13 year-olds.* Developmental Psychobiology, 2012. **54**(7): p. 675-684.
58. Lissek, S., et al., *Overgeneralization of conditioned fear as a pathogenic marker of panic disorder.* Am J Psychiatry, 2010. **167**(1): p. 47-55.
59. Hunt, M.G., L. Rodriguez, and E. Marcelle, *A cognitive behavioral therapy workbook delivered online with minimal therapist feedback improves quality of life for inflammatory bowel disease patients.* Internal Medicine Review, 2017. **3**(10): p. 1-16.
60. Klein, Z., et al., *Enhanced late positive potential to conditioned threat cue during delayed extinction in anxious youth.* Journal of Child Psychology and Psychiatry, 2023. **n/a**(n/a).

61. Klein, Z., et al., *The effects of age and trait anxiety on avoidance learning and its generalization.* Behaviour Research and Therapy, 2020. **129**: p. 103611.
62. Lee, J.C., B.K. Hayes, and P.F. Lovibond, *Peak shift and rules in human generalization.* Journal of Experimental Psychology: Learning, Memory, and Cognition, 2018. **44**(12): p. 1955-1970.
63. Li, X., Y. Yang, and X. Zheng, *Generalization of conditioned fear in humans: Transitioning from episodic to semantic memory.* Learning and Motivation, 2024. **88**: p. 102047.
64. Lissek, S., et al., *Elevated fear conditioning to socially relevant unconditioned stimuli in social anxiety disorder.* Am J Psychiatry, 2008. **165**(1): p. 124-32.
65. Manbeck, A.B., S.E. Cooper, and S. Lissek, *Reversing threat contingencies enhances generalization of conditioned fear.* Learning and Motivation, 2022. **80**: p. 101843.
66. Michalska, K.J., et al., *A developmental analysis of threat/safety learning and extinction recall during middle childhood.* Journal of Experimental Child Psychology, 2016. **146**: p. 95-105.
67. Niederstrasser, N.G., et al., *Executive functions deficits impair extinction of generalization of fear of movement-related pain.* European Journal of Pain, 2017. **21**(5): p. 886-899.
68. Phillips, L.W., *Mediated verbal similarity as a determinant of the generalization of a conditioned GSR.* Journal of Experimental Psychology, 1958. **55**(1): p. 56-62.
69. Reinhard, J., et al., *Fear conditioning and fear generalization in children and adolescents with anxiety disorders.* European Child & Adolescent Psychiatry, 2024. **33**(7): p. 2163-2172.
70. Roesmann, K., et al., *Developmental aspects of fear generalization – A MEG study on neurocognitive correlates in adolescents versus adults.* Developmental Cognitive Neuroscience, 2022. **58**: p. 101169.
71. Struyf, D., D. Hermans, and B. Vervliet, *Maximizing the generalization of fear extinction: Exposures to a peak generalization stimulus.* Behaviour Research and Therapy, 2018. **111**: p. 1-8.
72. Torrents-Rodas, D., et al., *No effect of trait anxiety on differential fear conditioning or fear generalization.* Biological Psychology, 2013. **92**(2): p. 185-190.
73. Vandael, K., et al., *The effect of experimentally induced positive affect on the generalization of pain-related avoidance and relief.* Behaviour Research and Therapy, 2023. **165**: p. 104324.
74. Vandael, K., et al., *The relationship between fear generalization and pain modulation: an investigation in healthy participants.* 2020. **20**(1): p. 151-165.
75. van Meurs, B., et al., *Maladaptive behavioral consequences of conditioned fear-generalization: A pronounced, yet sparsely studied, feature of anxiety pathology.* Behaviour Research and Therapy, 2014. **57**: p. 29-37.
76. Vervliet, B., D. Vansteenwegen, and P. Eelen, *Generalization Gradients for Acquisition and Extinction in Human Contingency Learning.* Experimental Psychology, 2006. **53**(2): p. 132-142.

77. Wong, A.H.K. and P.F. Lovibond, *Rule-based generalisation in single-cue and differential fear conditioning in humans.* Biological Psychology, 2017. **129**: p. 111-120.

78. Wong, A.H.K. and P.F. Lovibond, *Excessive generalisation of conditioned fear in trait anxious individuals under ambiguity.* Behaviour Research and Therapy, 2018. **107**: p. 53-63.

79. Wong, A.H.K., et al., *Generalization of extinction with a generalization stimulus is determined by learnt threat beliefs.* Behaviour Research and Therapy, 2020. **135**: p. 103755.

80. Wong, A.H.K. and A. Pittig, *Costly avoidance triggered by categorical fear generalization.* Behaviour Research and Therapy, 2020. **129**: p. 103606.

81. Wong, A.H.K., et al., *The degree of safety behaviors to a safety stimulus predicts development of threat beliefs.* Behaviour Research and Therapy, 2023. **170**: p. 104423.

82. Zoladz, P., et al. *Childhood Maltreatment in Females Is Associated with Enhanced Fear Acquisition and an Overgeneralization of Fear*. Brain Sciences, 2022. **12**, DOI: 10.3390/brainsci12111536.

83. Antov, M.I., et al., *Visuocortical tuning to a threat-related feature persists after extinction and consolidation of conditioned fear.* Scientific Reports, 2020. **10**(1): p. 3926.

84. Friedl, M.W. and A. Keil, *Aversive Conditioning of Spatial Position Sharpens Neural Population-Level Tuning in Visual Cortex and Selectively Alters Alpha-Band Activity.* The Journal of Neuroscience, 2021. **41**(26): p. 5723.

85. McTeague, L.M., L.F. Gruss, and A. Keil, *Aversive learning shapes neuronal orientation tuning in human visual cortex.* Nat Commun, 2015. **6**: p. 7823.

86. Plog, E., et al., *Phase-Synchronized Stimulus Presentation Augments Contingency Knowledge and Affective Evaluation in a Fear-Conditioning Task.* eneuro, 2022. **9**(1): p. ENEURO.0538-20.2021.

87. Stegmann, Y., et al., *Social aversive generalization learning sharpens the tuning of visuocortical neurons to facial identity cues.* eLife, 2020. **9**: p. e55204.

88. Ginat-Frolich, R., et al., *Reducing fear overgeneralization in children using a novel perceptual discrimination task.* Behaviour Research and Therapy, 2019. **116**: p. 131-139.

89. Ginat-Frolich, R., et al., *A novel perceptual discrimination training task: Reducing fear overgeneralization in the context of fear learning.* Behaviour Research and Therapy, 2017. **93**: p. 29-37.

90. Keefe, J.R., et al., *Elucidating behavioral and functional connectivity markers of aberrant threat discrimination in PTSD.* Depression and Anxiety, 2022. **39**(12): p. 891-901.

91. Lommen, M.J.J., et al., *Training discrimination diminishes maladaptive avoidance of innocuous stimuli in a fear conditioning paradigm.* PLOS ONE, 2017. **12**(10): p. e0184485.

92. Meulders, A., et al., *Extinction of Fear Generalization: A Comparison Between Fibromyalgia Patients and Healthy Control Participants.* The Journal of Pain, 2017. **18**(1): p. 79-95.
93. Dos Santos Corrêa, M., et al., *Relationship between footshock intensity, post-training corticosterone release and contextual fear memory specificity over time.* Psychoneuroendocrinology, 2019. **110**: p. 104447.
94. Gao, Y., et al., *Contingency Reversal in Conditioned Fear Learning: The Moderated Mediation Model of Intolerance of Uncertainty and Instruction.* Psychology Research and Behavior Management, 2024: p. 1007-1020.
95. Fan, M., et al., *Stimulus diversity increases category-based fear generalization and the effect of intolerance of uncertainty.* Behaviour Research and Therapy, 2022. **159**: p. 104201.
96. Nelson, B.D., et al., *An Event-Related Potential Investigation of Fear Generalization and Intolerance of Uncertainty.* Behav Ther, 2015. **46**(5): p. 661-70.
97. Schroijen, M., et al., *Generalization of Fear to Respiratory Sensations.* Behav Ther, 2015. **46**(5): p. 611-26.
98. Yu, K., et al., *Humans display interindividual differences in the latent mechanisms underlying fear generalization behaviour.* Communications Psychology, 2023. **1**(1): p. 5.
99. Zaman, J., et al., *Influence of Interoceptive Fear Learning on Visceral Perception.* Psychosomatic Medicine, 2016. **78**(2).
100. Kass, M.D. and J.P. McGann, *Persistent, generalized hypersensitivity of olfactory bulb interneurons after olfactory fear generalization.* Neurobiology of Learning and Memory, 2017. **146**: p. 47-57.
101. Kopp, B., M. Schlimm, and C. Hermann, *Memory–emotional interactions as revealed by fear generalization in animal-fearful individuals.* Journal of Behavior Therapy and Experimental Psychiatry, 2005. **36**(2): p. 145-166.
102. Liu, S.S., *Differential conditioning and stimulus generalization of the rabbit's nictitating membrane response.* Journal of Comparative and Physiological Psychology, 1971. **77**(1): p. 136-142.
103. Miasnikov, A.A., J.C. Chen, and N.M. Weinberger, *Behavioral memory induced by stimulation of the nucleus basalis: Effects of contingency reversal.* Neurobiology of Learning and Memory, 2009. **91**(3): p. 298-309.
104. Scarlata, M.J., et al., *Chemogenetic stimulation of the infralimbic cortex reverses alcohol-induced fear memory overgeneralization.* Scientific Reports, 2019. **9**(1): p. 6730.
105. Torrents-Rodas, D., et al., *Testing the temporal stability of individual differences in the acquisition and generalization of fear.* Psychophysiology, 2014. **51**(7): p. 697-705.

106. You, Y., et al., *Pattern differentiation and tuning shift in human sensory cortex underlie long-term threat memory.* Current Biology, 2022. **32**(9): p. 2067-2075.e4.

107. Dou, H., et al., *Intranasal oxytocin decreases fear generalization in males, but does not modulate discrimination threshold.* Psychopharmacology, 2021. **238**(3): p. 677-689.

108. Holt, D.J., et al., *A parametric study of fear generalization to faces and non-face objects: relationship to discrimination thresholds.* Frontiers in Human Neuroscience, 2014. **8**.

109. Levine, S.M., et al., *Supracategorical fear information revealed by aversively conditioning multiple categories.* Cognitive Neuroscience, 2021. **12**(1): p. 28-39.

110. Lim, S.-L. and L. Pessoa, *Affective learning increases sensitivity to graded emotional faces.* Emotion, 2008. **8**(1): p. 96-103.

111. Shalev, L., R. Paz, and G. Avidan, *Visual Aversive Learning Compromises Sensory Discrimination.* J Neurosci, 2018. **38**(11): p. 2766-2779.

112. Sterpenich, V., et al., *Sleep sharpens sensory stimulus coding in human visual cortex after fear conditioning.* NeuroImage, 2014. **100**: p. 608-618.

113. R Development Core Team, *R: A language and environment for statistical computing*. 2021, R Foundation for Statistical Computing: Vienna, Austria.

114. Laux, L. and C.D. Spielberger, *Das State-Trait-Angstinventar: STAI*. 1981: Beltz Weinheim.

115. Kemper, C.J., M. Ziegler, and S. Taylor, *Überprüfung der psychometrischen Qualität der deutschen Version des Angstsensitivitätsindex-3.* Diagnostica, 2009. **55**(4): p. 223-233.

116. Taylor, S., et al., *Robust dimensions of anxiety sensitivity: development and initial validation of the Anxiety Sensitivity Index-3.* Psychol Assess, 2007. **19**(2): p. 176-88.

117. Chambless, D.L., et al., *Assessment of fear of fear in agoraphobics: the body sensations questionnaire and the agoraphobic cognitions questionnaire.* J Consult Clin Psychol, 1984. **52**(6): p. 1090-7.

118. Ehlers, A., J. Margraf, and D. Chambless, *Fragebogen zu körperbezogenen Ängsten, Kognitionen und Vermeidung: AKV*. 2001: Beltz-Test.

119. Liebowitz, M.R., *Social phobia.* Modern Problems of Pharmacopsychiatry, 1987.

120. Stangier, U. and T. Heidenreich, *Die Liebowitz Soziale Angst-Skala (LSAS)[Liebowitz Social Anxiety Scale]. Collegium Internationale Psychiatriae Scalarum (Ed.), Internationale Skalen für Psychiatrie [International Psychiatry Scales]*. 2004, Weinheim: Beltz. German.

121. Beidel, D.C., et al., *The social phobia and anxiety inventory: Concurrent and external validity.* Behavior Therapy, 1989. **20**(3): p. 417-427.

122. Bernstein, D.P., et al., *Development and validation of a brief screening version of the Childhood Trauma Questionnaire.* Child Abuse Negl, 2003. **27**(2): p. 169-90.

123. Wingenfeld, K., et al., *[The German version of the Childhood Trauma Questionnaire (CTQ): preliminary psychometric properties].* Psychother Psychosom Med Psychol, 2010. **60**(11): p. 442-50.

124. Lau, J.Y., et al., *Fear conditioning in adolescents with anxiety disorders: results from a novel experimental paradigm.* J Am Acad Child Adolesc Psychiatry, 2008. **47**(1): p. 94-102.

125. Boucsein, W., et al., *Publication recommendations for electrodermal measurements.* Psychophysiology, 2012. **49**(8): p. 1017-34.
